# Differences in Gut Microbiota Profiles and Microbiota Steroid Hormone Biosynthesis in Men with and Without Prostate Cancer

**DOI:** 10.1016/j.euros.2024.02.004

**Published:** 2024-03-13

**Authors:** Sofia Kalinen, Teemu Kallonen, Marianne Gunell, Otto Ettala, Ivan Jambor, Juha Knaapila, Kari T. Syvänen, Pekka Taimen, Matti Poutanen, Hannu J. Aronen, Helena Ollila, Sami Pietilä, Laura L. Elo, Tarja Lamminen, Antti J. Hakanen, Eveliina Munukka, Peter J. Boström, the Multi-IMPROD Study Group

**Affiliations:** a Research Center for Infections and Immunity, Institute of Biomedicine, University of Turku, Turku, Finland; b Department of Clinical Microbiology, Turku University Hospital, Turku, Finland; c Clinical Microbiome Bank, Microbe Center, Turku University Hospital and University of Turku, Turku, Finland; d Department of Urology, Turku University Hospital and University of Turku, Turku, Finland; e Department of Diagnostic Radiology, Turku University Hospital and University of Turku, Turku, Finland; f Institute of Biomedicine, University of Turku, Turku, Finland; g Department of Pathology, Turku University Hospital, Turku, Finland; h Centre for Integrative Physiology and Pharmacology, University of Turku, Turku, Finland; i Department of Internal Medicine and Clinical Nutrition, Institute of Medicine, Sahlgrenska Academy, University of Gothenburg, Gothenburg, Sweden; j Turku Clinical Research Centre, Turku University Hospital, Turku, Finland; k Turku Bioscience Centre, University of Turku and Åbo Akademi University, Turku, Finland; l Biocodex: Biocodex Nordics, Espoo, Finland; m Enterprise Service Group - Radiology, Mass General Brigham, Boston, MA

**Keywords:** Gut microbiota, Prostate cancer

## Abstract

**Background:**

Although prostate cancer (PCa) is the most common cancer in men in Western countries, there is significant variability in geographical incidence. This might result from genetic factors, discrepancies in screening policies, or differences in lifestyle. Gut microbiota has recently been associated with cancer progression, but its role in PCa is unclear.

**Objective:**

Characterization of the gut microbiota and its functions associated with PCa.

**Design, setting, and participants:**

In a prospective multicenter clinical trial (NCT02241122), the gut microbiota profiles of 181 men with a clinical suspicion of PCa were assessed utilizing 16S rRNA sequencing.

**Outcome measurements and statistical analysis:**

Sequences were assigned to operational taxonomic units, differential abundance analysis, and α- and β-diversities, and predictive functional analyses were performed. Plasma steroid hormone levels corresponding to the predicted microbiota steroid hormone biosynthesis profiles were investigated.

**Results and limitations:**

Of 364 patients, 181 were analyzed, 60% of whom were diagnosed with PCa. Microbiota composition and diversity were significantly different in PCa, partially affected by *Prevotella 9*, the most abundant genus of the cohort, and significantly higher in PCa patients. Predictive functional analyses revealed higher 5-α-reductase, copper absorption, and retinol metabolism in the PCa-associated microbiome. Plasma testosterone was associated negatively with the predicted microbial 5-α-reductase level.

**Conclusions:**

Gut microbiota of the PCa patients differed significantly compared with benign individuals. Microbial 5-α-reductase, copper absorption, and retinol metabolism are potential mechanisms of action. These findings support the observed association of lifestyle, geography, and PCa incidence.

**Patient summary:**

In this report, we found that several microbes and potential functions of the gut microbiota are altered in prostate cancer compared with benign cases. These findings suggest that gut microbiota could be the link between environmental factors and prostate cancer.

## Take Home Message

Gut microbiota of the prostate cancer patients is altered significantly compared with that of benign individuals. Microbial 5-α-reductase, copper absorption, and retinol metabolism are potential mechanisms of action. These findings support the previously observed association of lifestyle, geography, and prostate cancer incidence.

## 1 Introduction

Prostate cancer (PCa), despite its high incidence, has undiscovered details of etiology and pathogenesis [1], [2]. PCa is known to be highly heritable, but lifestyle, socioeconomic, and environmental factors may also affect PCa incidence [3]. Diet is one of the most widely studied lifestyle factors, and various nutrients and food products have been reported to be associated with an altered PCa risk [3], [4].

PCa incidence differs markedly between geographical locations, being the lowest in Asia and the highest in the Western lifestyle countries [2]. Differences in ethnicity, genetics, as well as healthcare-related factors such as intensity of prostate-specific antigen (PSA) screening have a significant effect on the reported variability of global PCa incidence [3]. However, other explanatory factors might also exist. It is of great interest that a high rate of incidental PCa has also been reported widely in the geographical areas of a low clinical PCa prevalence [5]. Additionally, studies conducted in immigrant populations suggest that nongenetic, individual lifestyle factors may affect PCa risk significantly [6]. Based on these observations, one might expect that prostate carcinogenesis affects a significant portion of aging men, if not all of them. One might also expect that individual lifestyle and environmental factors may either stimulate or inhibit the neoplastic process in the prostate, accounting for the geographical differences observed in clinical PCa. However, the mechanisms mediating how lifestyle affects PCa risk remains unclear.

Gut microbiota (GM), that is, a collection of all microbes in the gastrointestinal tract, is considered to affect many metabolic pathways and pathogenetic processes in the human body [7]. Furthermore, gut dysbiosis (disequilibrium of the microbiota) leading to low-grade inflammation has been linked to many cancers, also in organs distant from the intestines [7]. Chronic inflammation, production of superoxide radicals, growth factors, and bacterial genotoxins have all been proposed as mechanisms of action [7]. Furthermore, the alterations in GM could result from the lifestyle factors related to the altered PCa risk.

A majority of the PCa investigations covering microbiological aspects have studied either prostate tissue or urinary tract microbiota with conflicting results [8]. To date, the effect of GM on prostate carcinogenesis is documented poorly, although there is some evidence of an association with PCa [8], [9]. The association of fecal microbiota with PCa has received little attention, and most studies have very small sample sizes [8]. Larger studies to date reported differences in GM composition between PCa and non-PCa cases, as well as between high-risk and benign low-risk PCa [9], [10], [11]. Microbiome analyses also suggested mechanisms of action, including altered folate and arginine metabolism, as well as short-chain fatty acids and IGF-1 signaling [9], [10], [12].

To assess the fecal microbiota profiles of PCa patients compared with their benign counterparts, we conducted a substudy within a prospective clinical trial (NCT02241122) where microbiological swab samples from men with suspected PCa were 16S sequenced, sequences were analyzed with bioinformatic methods, and steroid hormone levels were measured from plasma. To our knowledge, this is the largest and most detailed clinical trial studying the GM of PCa patients.

## 2 Patients and methods

### 2.1 Trial design

The study cohort has previously been reported in detail [13]. Between February 2015 and March 2017, men with a clinical suspicion of PCa were enrolled at four Finnish hospitals for a prospective, investigator-initiated, open-label, nonrandomized trial investigating magnetic resonance imaging (MRI) and biomarkers in PCa diagnosis (Clinicaltrials.gov, NCT02241122). Informed consent was obtained from all study participants. The study protocol, patient information sheet, and informed consent forms were approved by the Ethics Committee of the Hospital District of Southwest Finland (no. 6/2014). The ethical principles of the Declaration of Helsinki (59th World Medical Association General Assembly, Seoul, Korea, 2008) were followed.

### 2.2 Patients

All men included were clinically suspected of having PCa, with PSA ranging from 2.5 to 20.0 μg/l and/or an abnormal finding in digital rectal examination (DRE). The exclusion criteria included previous prostate biopsy, previous prostate surgery, previous diagnosis of PCa, acute prostatitis, or contraindications for MRI.

### 2.3 Assessments

After MRI, 12-core systematic and two targeted transrectal biopsies from up to two lesions suspected at MRI (Likert score 3–5) were collected from all participants. Prophylactic antibiotics were given according to the institutional guidelines and have previously been reported in detail [14]. In practice, patients received either single or two doses of antibiotics. The distribution of received specific antibiotics was 60% ciprofloxacin, 30% levofloxacin, and 2% fosfomycin. No enema was administered prior to biopsy. Immediately prior to transrectal biopsies, microbiome samples were collected utilizing sterile rectal swabs (Copan FLOQSwab; Copan Diagnostics Inc., Murrieta, CA, USA) and immediately stored at –20 °C. Blood samples were collected for plasma steroid assays. In addition to clinical specimens, a detailed questionnaire including a family history of PCa, and general medical, travel, and smoking histories was completed prior to biopsy.

### 2.4 Microbiological sample preparation for 16S RNA sequencing

Stool samples were diluted in 1 ml of sterile saline after which 500 µl was used for DNA extraction with the semiautomatic GXT Stool Extraction Kit VER 2.0 and GenoXtract unit (Hain Lifescience GmbH, Nehren, Germany). DNA concentrations were measured with the Qubit dsDNA HS Assay Kit and Qubit 2.0 fluorometer (Life Technologies, Carlsbad, CA, USA). Extracted DNA was stored at –80 °C until use. Bacterial V4 gene regions of 16S rRNA were sequenced in three batches with MiSeq (Illumina, San Diego, CA, USA), including aqua as negative control, and seven ATCC strain 16S rRNA genes in plasmids (*Bifidobacterium adolescentis, Escherichia coli, Enterococcus faecalis, Faecalibacterium prausnitzii, Lactobacillus acidophilus, Staphylococcus epidermidis,* and *Streptococcus pyogenes*). This in-house method has previously been described in detail [15].

### 2.5 Data analysis and bioinformatics

Statistical analyses were performed with the IBM Statistical Package for the Social Sciences (SPSS) for Windows, version 26, 64-bit (IBM Corp., Armonk, NY, USA) and R version 4.0.3. Continuous variables were summarized with medians and quartiles. For the main study questions, difference of medians and 95% confidence intervals (CIs) were calculated with quantile regression. PCa cases were grouped by two methods, each utilizing the International Society of Urological Pathology (ISUP) grade, namely, benign cases versus all cancer cases, and benign cases versus ISUP grade group 1, 2–3, and 4–5 cancers [16]. Factors potentially impacting either cancer status or GM composition, such as age, body mass index, recent use of antibiotics, inflammatory bowel diseases (IBDs), recent high-risk travel history, and smoking, were also analyzed with Wilcoxon rank sum, Kruskal-Wallis, Pearson chi-square, or Fisher’s exact tests.

Microbial analyses were performed with CLC Genomics Workbench Microbial Genomics module v. 12 (QIAGEN Digital Insights, Aarhus, Denmark). Sequences were assigned to operational taxonomic units (OTUs) according to the similarity of sequences with CLC Microbial Genomics module workflow. Quality and ambiguous trims were performed with default settings, with the minimum number of nucleotides set to 150. SILVA 16S v132 97% was used as the reference database [17], [18].

Microbial diversity was defined with α-diversity that measures diversity within one sample, and β-diversity that measures similarity or dissimilarity of two distinct samples. The α-diversity indices Chao1 and Shannon were calculated to evaluate community richness, diversity, and evenness at 10527-rarefaction level. A β-diversity measure, Bray-Curtis, was calculated to assess compositional dissimilarity of two samples between PCa and benign, the statistical significance of which was evaluated with the permutational multivariate analysis of variance (PERMANOVA) test with 99 999 permutations. The sequencing batch effect was controlled with visualization of β-diversity measures (Bray-Curtis and UniFrac) and PERMANOVA tests.

A differential abundance analysis with a generalized linear model that applies a negative binomial distribution was also performed. The sequencing batch and the previously mentioned factors potentially impacting GM were corrected in the analysis. Age was not considered in the analysis because of collinearity with PCa status. The results were filtered with combined abundance in all samples of >100 and a prevalence of ≥10%. The *p* values were corrected using the false discovery rate (FDR) approach [19]. The statistical significance limit was set at *p* < 0.05.

A predictive analysis of functional bacterial genes was constructed utilizing Phylogenetic Investigation of Communities by Reconstruction of Unobserved States (PICRUSt) v.1.0 tool [20]. PICRUSt was performed with Qiime v. 1.9 to create Kyoto Encyclopedia of Genes and Genomes (KEGG) orthologs and pathways according to the counts of OTUs with the reference data of Greengenes v.13.8 [21], [22], [23]. As microbiota and predicted gene products were not normally distributed, data were analyzed with nonparametric Wilcoxon rank sum and Kruskal-Wallis tests, and described with medians and 95% CIs.

### 2.6 Plasma steroid assay

The steroid analyses of plasma samples were conducted using liquid chromatography-tandem mass spectrometry according to an established method [24]. The measured steroids included androstenedione, dehydroepiandrosterone, dihydrotestosterone (DHT), estradiol, estrone, progesterone, 17-α-hydroxyprogesterone, and testosterone (T). In addition, the DHT/T ratio was calculated for statistical analyses. Wilcoxon rank sum tests and Spearman correlations were used to investigate the association of PICRUSt-predicted microbial steroid hormone biosynthesis with plasma steroid status. For Wilcoxon rank sum tests, steroid hormone biosynthesis was divided into high and low according to the median, to compare hypothesized low/normal and high/pathological values.

## 3 Results

### 3.1 Study participants

**Fig. 1:**
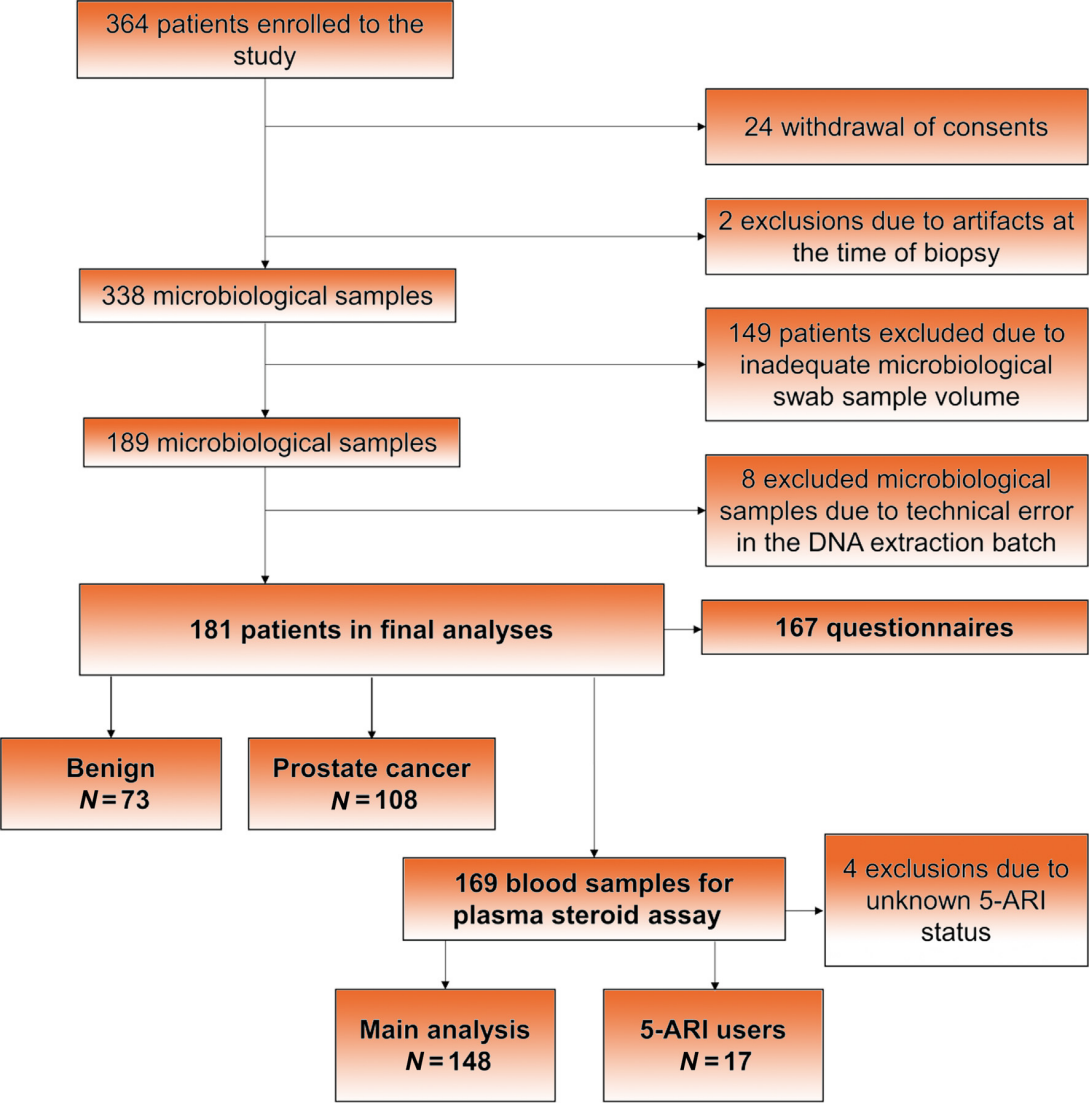
Study flowchart. A total of 364 patients were enrolled in the study, and after exclusion, 181 were included in the analyses. The available 169 blood samples were divided according to their use of 5-α-reductase inhibitors (5-ARI) into the main analysis group and 5-ARI users’ group.

A total of 364 men entered the trial (see the flowchart in Fig. 1). After excluding patients withdrawing consent (*n* = 24), having MRI artifacts at the time of biopsy (*n* = 2), having stool material in rectal swabs insufficient for analyses (*n* = 149), and being in the technically unsuccessful DNA extraction batch (*n* = 8; Supplementary Fig. 1), a total of 181 men were included in the analyses, of whom 167 provided complete questionnaires. Plasma samples were available from 169 cases, of which four were excluded due to the unknown status of 5-α-reductase inhibitor (5-ARI) medication. Plasma steroids of 5-ARI users were analyzed separately (*n* = 17, 10%).

The basic clinicopathological characteristics and the most significant confounding factors potentially affecting microbiota composition are presented in Table 1. Age, PSA, PSA density, and prostate volume were significantly associated with cancer status and ISUP cancer grade. PCa was diagnosed in 60% and clinically significant PCa in 46% of cases (respectively, 108/181 and 207/338, 61% PCa/43% clinically significant PCa for the whole cohort). Of the 108 diagnosed PCa cases, 23% were ISUP grade 1, 45% ISUP grade 2–3, and 31% ISUP grade 4–5. Potentially confounding factors were similar between groups except that the PCa group contained fewer active smokers (7%) than the benign group (13%). Smoking was not associated with cancer grade (Table 1).

**Table 1:** Clinicopathological characteristics of the participants

Characteristic	*N*	Benign (*N* = 73)		Prostate cancer (*N* = 108)		Prostate cancer vs benign *p* value	Prostate cancer						Across the grade groups vs benign *p* value a
							ISUP grade 1 (*N* = 25)		ISUP grade 2–3 (*N* = 49)		ISUP grade 4–5 (*N* = 34)		
		Median	Q1-Q3	Median	Q1-Q3		Median	Q1-Q3	Median	Q1-Q3	Median	Q1-Q3	
Age (yr)	167	62	55–67	68	62–72	**<0.001**^b^	63	58–68	68	65–73	69	66–72	**<0.001**^a^
BMI (kg/m^2^)	164	26.0	24.1–29.5	27.1	25.1–29.0	0.19 b	26.9	24.7–28.9	26.6	24.1–29.2	27.5	26.0–29.0	0.36 a
PSA (µg/l)	181	6.1	4.3–8.0	7.9	5.9–10.9	**<0.001**^b^	6.0	4.1–9.9	8.4	7.0–10.0	8.0	5.8–11.3	**<0.001**^a^
PSA density (ng/ml2)	175	0.12	0.10–0.20	0.21	0.15–0.30	**<0.001**^b^	0.14	0.10–0.26	0.24	0.16–0.32	0.23	0.18–0.28	**<0.001**^a^
Free PSA ratio (%)	155	16	11–20	13	11–18	0.21 b	16	11–21	14	11–19	12	10–16	0.19 a
Prostate volume (ml)	175	44	33–62	36	29–48	**0.003**^b^	39	31–49	35	28–52	36	31–43	**0.031**^a^
		%		%			%		%		%		
5-α-reductase medication c	167	12		9		0.46 d	8		4		18		0.18 e
Antibiotic treatments within a year	165	32		28		0.55 d	30		20		39		0.30 d
Active smoking	165	20		9		**0.042**^d^	13		9		7		0.20 d
Special diet	165	2		3		1.000 e	8		2		0		0.26 e
High-risk traveling within a year f	167	6		6		1.000 e	4		9		3		0.83 e
Inflammatory bowel disease	167	5		0		0.060 e	0		0		0		0.34 e

BMI = body mass index; ISUP = International Society of Urological Pathology; PSA = prostate-specific antigen.

a Kruskal-Wallis test.

b Wilcoxon rank sum test.

c Finasteride/dutasteride.

d Pearson chi-square test.

e Fisher's exact test.

f High risk for antimicrobial resistance (Africa, Asia, and South America).

### 3.2 Gut microbial diversity

**Fig. 2:**
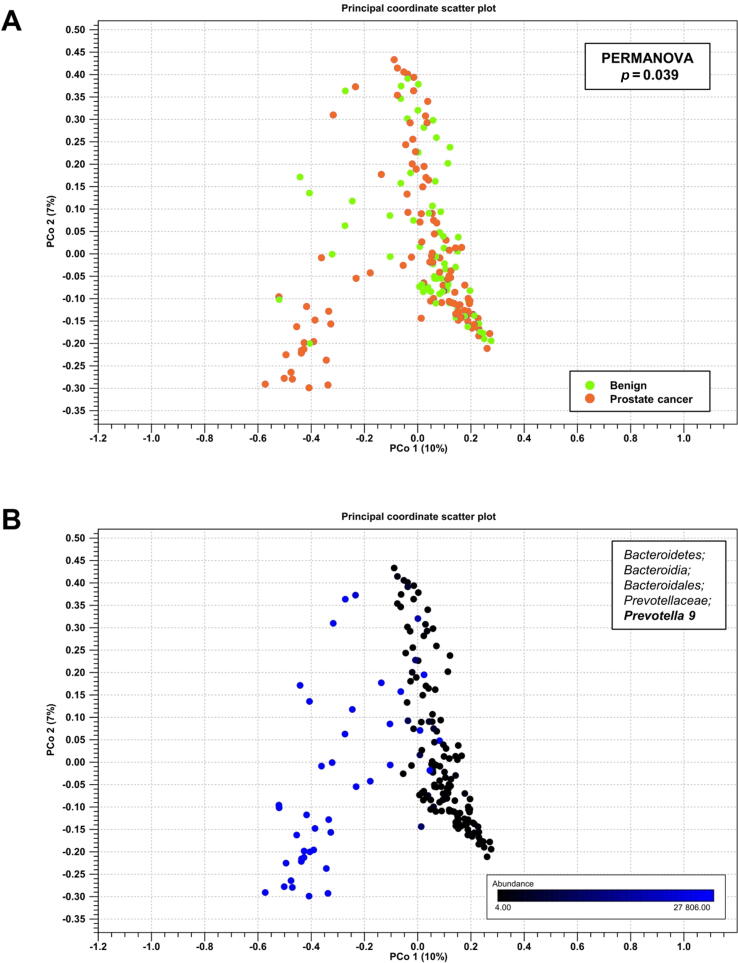
(A) Bray-Curtis dissimilarity (β-diversity) of the gut microbiota between benign and prostate cancer (PCa) cases. Benign cases are highlighted with green and PCa cases with purple dots. The microbiota of benign and cancer patients differs significantly (PERMANOVA pseudo f-statistic 1.5, *p* = 0.039). (B) Abundance of *Prevotella 9* in the principal coordinate (PCo) scatter plot 1 (10% of the calculated differences in diversity). Abundance of *Prevotella 9* is also significantly elevated in PCa (differential abundance analysis Log2 fold change 1.5, FDR corrected *p* = 0.03). The color scale represents the abundance of *Prevotella 9*, varying from 4 (black) to 27 806 (blue). Upper bound on the color scale is Q3 (27 806) of the prostate cancer cases. FDR = false discovery rate; PERMANOVA = permutational multivariate analysis of variance.

According to the α-diversity indices Chao1 (*p* = 0.7) and Shannon (*p* = 0.3), no significant differences were found in bacterial community richness or evenness (Supplementary Fig. 2–4). On the contrary, compositional dissimilarity differed between PCa cases and men without PCa (PERMANOVA *p* = 0.039; Fig. 2A), but these differences were not reflected in the cancer grade (PERMANOVA *p* = 0.23; Supplementary Fig. 5). Principal coordinate 1 in the Bray-Curtis analysis appears to consist of mostly *Prevotella 9* (10%; Fig. 2B), which was the most abundant single genus in the GM and associated with PCa in the differential abundance analysis.

### 3.3 GM composition and abundance

**Fig. 3:**
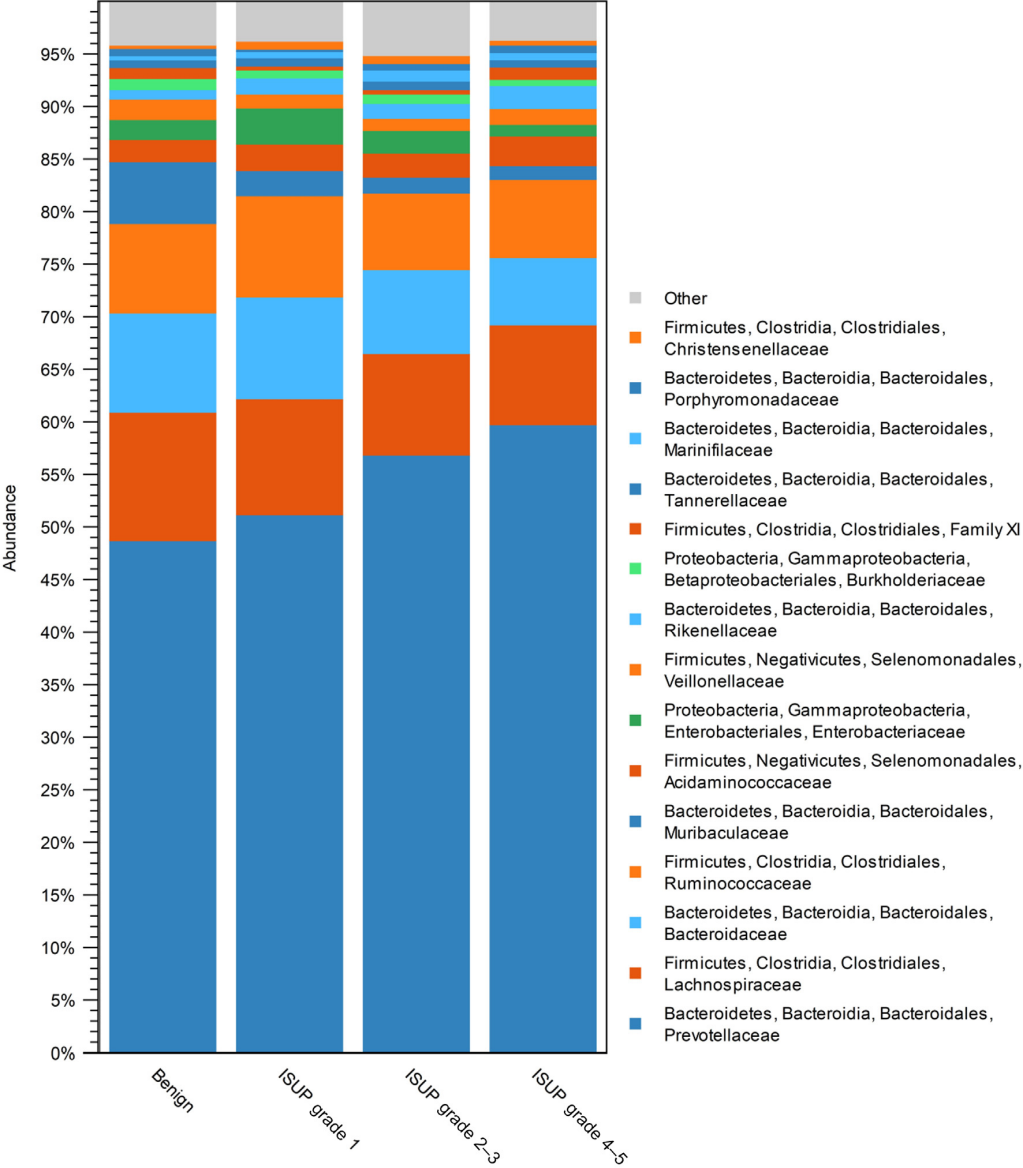
Relative abundances of the core microbiome level across the cancer grades at the family level. Bars indicate the percentage of the taxa. Color indicates the taxonomical phylum of the family: blue shades for *Bacteroidetes*, orange for *Firmicutes*, green for *Proteobacteria*, and gray for others. Prostate cancer cases were graded according to severity with ISUP grade groups. Family *Prevotellaceae* abundance elevates with cancer severity, but the elevation was not statistically significant (*p* = 0.86). ISUP = International Society of Urological Pathology.

**Fig. 4:**
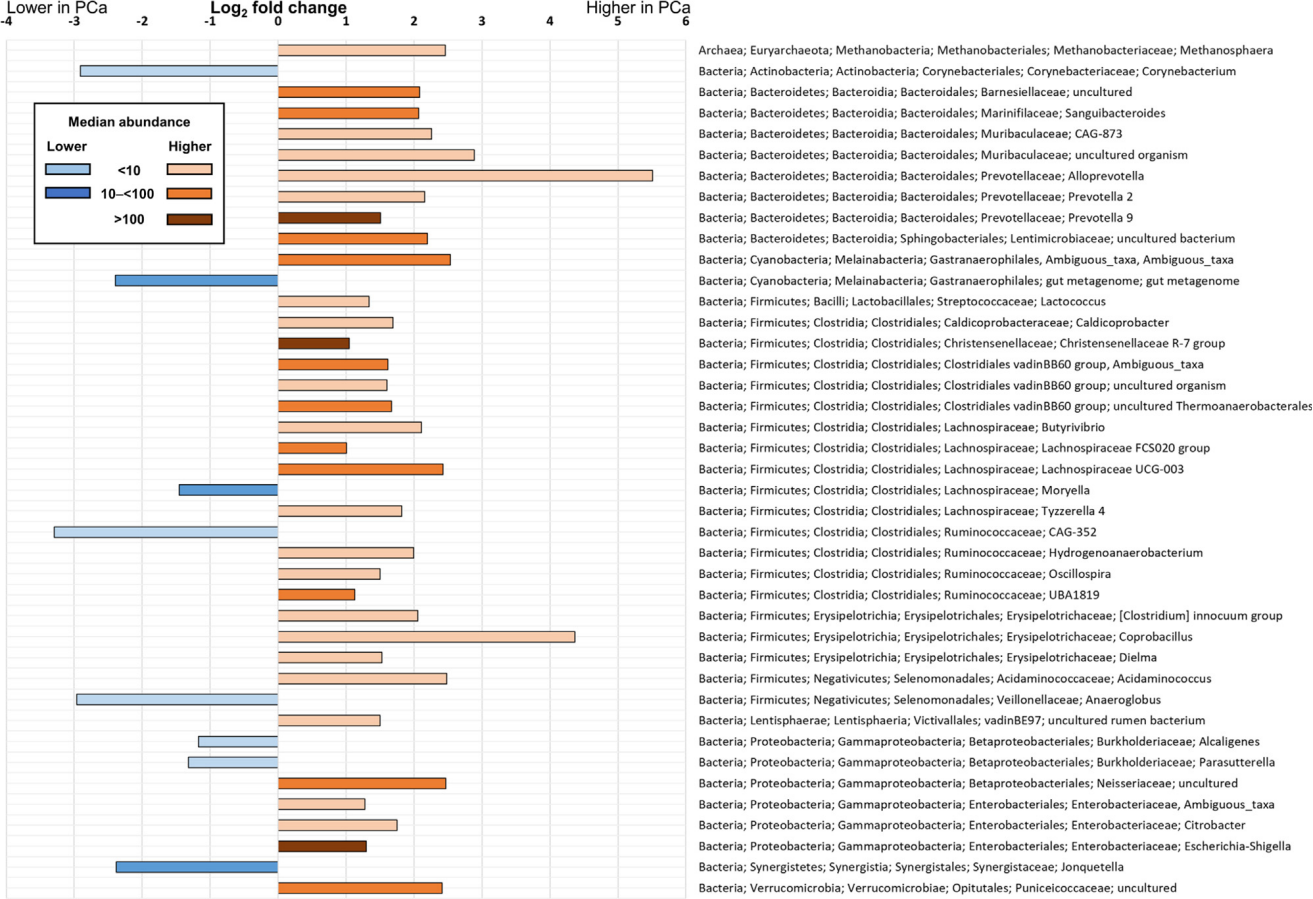
Statistically significant Log2 fold changes of microbial genera in prostate cancer versus benign. Genera more abundant in prostate cancer cases are shown with peach-orange-brown shaded bars, and genera lower in the cancer cases are shown with blue shaded bars. The shade of the color indicates the dominance of the genus by median abundance in the whole cohort. Genera are presented in taxonomical order. While *Prevotella 9*, *Christensenellaceae R-7*, and *Escherichia-Shigella* are abundant members of the gut microbiota, less abundant groups are also different. PCa = prostate cancer.

The 181 successfully sequenced samples contained bacterial taxa from 21 different phyla. At the family level, OTU clustering showed *Prevotellaceae* becoming gradually more abundant with increasing cancer grade, but the elevation was not statistically significant in the differential abundance analysis (max group mean 144 000, ISUP grade 4–5 vs benign Log2 fold change 0.61, *p* = 0.86; Fig. 3). Furthermore, differential abundance analysis showed that *Alloprevotella, Prevotella 2*, and *Prevotella 9* within the family *Prevotellaceae* were more abundant in the cancer group. Other significantly abundant genera belonged to *Acidaminococcaceae, Christensenellaceae*, *Clostridiales vadinBB60*, *Corynebacteriaceae*, *Enterobacteriaceae*, *Erysipelotrichaceae, Lachnospiraceae*, *Muribaculaceae, Ruminococcaceae*, *Synergistaceae,* and *Veillonellaceae* families. Genera higher and lower in PCa are presented in Figure 4 (Supplementary Table 1). The microbial taxa, which had significantly different abundance in the cancer and benign groups, had variable abundance correlation with the different PCa ISUP grades. For example, some microbes were especially abundant in low-grade cancers (eg, *Acidaminococcus*), while others were higher in men with higher-grade cancers (eg, *UBA1819, [Clostridium] innocuum*; Supplementary Fig. 6A–C).

### 3.4 Prediction of the GM functions

Three KEGG pathways were significantly different between the cancer and benign groups; mineral absorption (copper chaperone, difference of the medians 1570, 95% CI –1590, 4730, Wilcoxon rank sum *p* = 0.008), steroid hormone biosynthesis (5-α-reductase [5-AR], difference of the medians 3380, 95% CI –768, 7530, Wilcoxon rank sum *p* = 0.022), and retinol metabolism (difference of the medians 1630, 95% CI –4530, 7790, Wilcoxon rank sum *p* = 0.042) had significantly higher values in the PCa group than in men without cancer (Table 2 and Supplementary Table 2).

**Table 2:** PICRUSt-predicted statistically significant bacterial gene products associated with prostate cancer status

KEGG pathway	KEGG orthologs	Benign		Prostate cancer		Wilcoxon rank sum *p* value
		Median	95% CI	Median	95% CI	
Mineral absorption	Copper chaperone (95%)	2522	(1633; 3716)	4044	(2325; 6090)	**0.008**
	Heme oxygenase 1 (5%)					
Steroid hormone biosynthesis	5-α-reductase 1 (45%)	8457	(4514; 10 952)	11 717	(8508; 14 660)	**0.022**
	5-α-reductase 2 (54%)					
	3-α-hydroxysteroid hydrogenase (1%)					
Retinol metabolism	Alcohol dehydrogenase (67%)	21 605	(18 680; 23 784)	23 174	(21 171; 26 117)	**0.042**
	Alcohol dehydrogenase propanol preferring (5%)					
	All-trans-retinol 13,14-reductase (20%)					
	S-(hydroxymethyl) glutathione dehydrogenase/alcohol dehydrogenase (8%)					

CI = confidence interval; KEGG = Kyoto Encyclopedia of Genes and Genomes; PICRUSt = Phylogenetic Investigation of Communities by Reconstruction of Unobserved States.

### 3.5 Plasma steroids

**Fig. 5:**
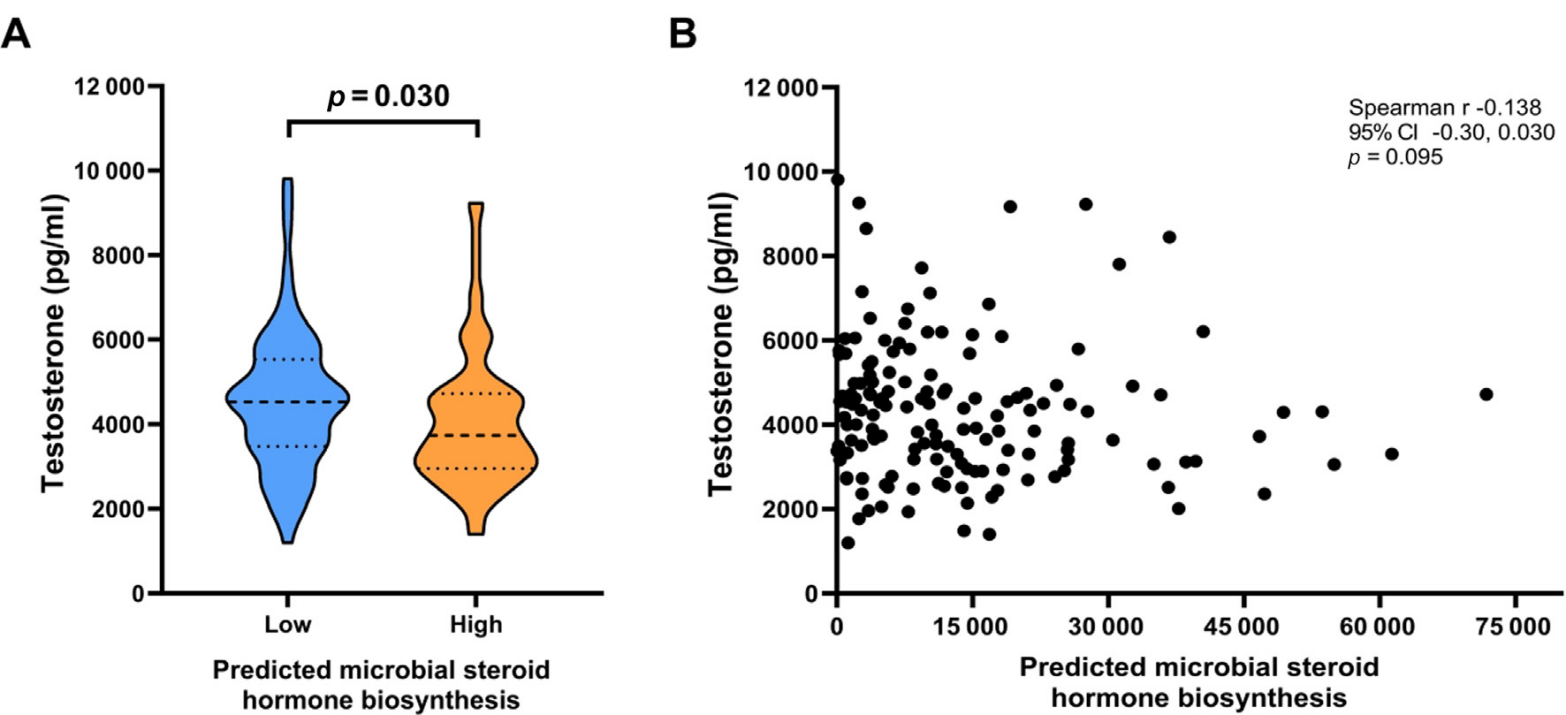
Testosterone concentration (pg/ml) in plasma by predicted microbial steroid hormone biosynthesis. (A) Plasma testosterone levels are significantly lower (Wilcoxon rank sum *p* = 0.03, *n* = 148) in patients who have high (above median) microbial steroid hormone biosynthesis without adjusting for the cancer status. (B) Plasma testosterone levels did not significantly correlate negatively (Spearman *p* = 0.095). CI = confidence interval.

The possible systemic effects of steroid hormone metabolism suggested by the KEGG pathways (PICRUSt) were investigated. First, among 5-ARI users (*n* = 17), the plasma DHT (difference of the medians 295, 95% CI 223, 366, *p* < 0.0001) and DHT/T ratio (difference of the medians 0.071, 95% CI 0.059, 0.082, *p* = 0.0002) were significantly lower than among nonusers, as expected (Supplementary Fig. 7). Among non–5-ARI users (*N* = 148), plasma T levels were negatively associated with the predicted microbial 5-AR (Spearman correlation –0.138, *p* = 0.095, difference of the medians 809, 95% CI 199, 1420, Wilcoxon rank sum *p* = 0.030; Fig. 5). Other measured hormone levels were not significantly altered according to microbial 5-AR, and there were no significant differences according to the PCa status (Supplementary Fig. 8 and 9).

## 4 Discussion

This study evaluated potential links between GM and PCa using GM profiles of 181 patients with suspected PCa. The 108 patients in whom PCa was diagnosed had significant differences in their GM signatures compared with the remaining 73 patients. Predictive functional analyses suggest that differences in the steroid hormone, copper, and retinol pathways could be the metabolic consequences of the altered microbiota. The hormone dependency of PCa could render it susceptible to alterations in steroid hormones caused by GM, which were also studied. Men having a predicted alteration in gut steroid hormone metabolism, measured as predicted higher microbial 5-AR, also had a pattern of plasma steroid concentration that differed from those not having this predicted elevated level.

The detailed differential abundance analysis revealed several alterations in the abundant and minor members of GM. The most abundant genus of the whole cohort, *Prevotella 9,* was also elevated in PCa. The family *Prevotellaceae* has been shown to be affected especially by diet since it is able to degrade complex plant polysaccharides, but different genera of the family may have distinct functions in promoting disease or supporting health [25]. Another abundant member, *Escherichia-Shigella* from the *Enterobacteriaceae* family, includes the opportunistic pathogen *E. coli* that has colibactin-mediated genotoxic properties and has been reported in PCa patients previously [7], [10]. By contrast, members of the *Erysipelotrichaceae* family have been suggested to play a role in the pathogenesis of several diseases, such as IBDs and metabolic disorders, despite being usually a minor member of the GM community [26]. Similarly to our results, *Christensenellaceae* was elevated and *Moryella* had a negative correlation with high-risk PCa in a previous report [11]. To conclude, several microbes higher in PCa have been linked to PCa as well as other diseases, highlighting the potential disease promoting properties of GM. However, divergent, and even contradictory, results compared with other studies could be caused by high variability between individuals, diverse pooling of groups, or statistical methods.

It is of great interest that the PICRUSt analysis indicated elevated steroid hormone biosynthesis pathways in the GM of the cancer cases, since it is well known that 5-AR reduces T to DHT feeding PCa. Formerly, higher steroid hormone biosynthesis has been discovered in the microbiota of patients with androgen deprivation therapy (*n* = 9) than in men without therapy (*n* = 16) [27]. Furthermore, the contribution of GM to the rate of PCa tumor growth and progression of castration resistance through steroid hormones in animal models as well as in human samples were shown recently [28]. These studies suggest that steroid metabolism of GM is associated with therapy response. However, our study suggests that microbiota-enriched 5-AR activity may have more profound effects in prostate carcinogenesis. In addition, we demonstrated that the systemic steroid hormone levels were associated with altered predicted microbial 5-AR. Moreover, we have recently shown high concentrations of DHT in the gut of mice models as well as in human individuals, with GM being responsible for the metabolic activity [29]. The potential mechanistic explanation for the altered steroid hormone metabolism caused by GM clearly warrants further studies.

The other potential metabolic pathways noted in our study were copper and retinol metabolisms that are less studied in PCa. Copper chaperone is a protein that ferries copper to cellular organelles, and interestingly, accumulation of copper has been reported in tumor cells, including PCa cells [30]. Androgen receptor activation may enhance copper uptake, again suggesting that hormonal pathways would be involved in altered GM metabolism [31]. In line with our findings, higher retinol concentrations have been associated with an elevated PCa risk, and genetic variants in retinol pathways have previously been associated with PCa [32].

The main limitation of our study is the lack of sample size estimations and power calculations limiting analyses of minor microbial groups, but this would have been impossible to carry out without prior studies. Furthermore, low stool content in the sampling swabs, resulting in an unsatisfactory amount of bacterial DNA for 16S rRNA sequencing in 149 of 338 samples, could have been avoided by the fecal sampling method. In addition, patients with recent antibiotic use were not excluded, even though antimicrobials could affect GM. To account for this, use of antibiotics was one of the variables used to correct the analyses. One should also note that the study was limited to individuals with a relatively low-risk cancer suspicion, which may potentially dilute the results as cases with advanced or metastatic tumors were not included. The strengths of our study include the prospective design, detailed prospective data collection, and that, to our knowledge, this is the largest reported study on the subject.

## 5 Conclusions

In this study, we discovered differences in GM components between PCa patients and benign individuals. In a predictive analysis, microbial steroid hormone biosynthesis, mineral absorption, and retinol metabolism were potential carcinogenic pathways. Moreover, elevated predicted microbial 5-AR was associated with lower T levels in plasma. These findings could explain the previously observed association of lifestyle, geography, and PCa incidence.

  ***Author contributions*:** Peter J. Boström had full access to all the data in the study and takes responsibility for the integrity of the data and the accuracy of the data analysis.

  *Study concept and design*: Boström, Munukka, Hakanen, Kallonen, Kalinen.

*Acquisition of data*: Kalinen, Munukka, Kallonen, Gunell, Ettala, Knaapila, Jambor, Syvänen, Taimen.

*Analysis and interpretation of data*: Kalinen, Kallonen, Munukka, Gunell, Boström, Poutanen, .

*Drafting of the manuscript*: Kalinen, Boström, Munukka, Hakanen, Kallonen.

*Critical revision of the manuscript for important intellectual content*: Boström, Kallonen, Munukka, Hakanen, Poutanen.

*Statistical analysis*: Kalinen, Kallonen, Munukka, Ollila, Pietilä.

*Obtaining funding*: Boström, Aronen.

*Administrative, technical, or material support*: Boström, Lamminen, Hakanen, Aronen, Elo, Poutanen, Ohlsson, Kallonen, Ollila, Montoya Perez.

*Supervision*: Boström, Munukka, Kallonen.

*Other*: All authors equally (review and editing).

  ***Financial disclosures***: Peter J. Boström certifies that all conflicts of interest, including specific financial interests and relationships and affiliations relevant to the subject matter or materials discussed in the manuscript (eg, employment/affiliation, grants or funding, consultancies, honoraria, stock ownership or options, expert testimony, royalties, or patents filed, received, or pending), are the following: Sofia Kalinen: grant from Finnish Cultural Foundation, University of Turku, and Federal grants, and a travel grant for WOM23 from Turun Mikrobiologien Tiedesäätiö. Hannu J. Aronen: grants of Special State Support for Clinical Research in Finland. Peter J. Boström: grants from Finnish Cancer Society, University of Turku, and Federal grants; consulting fees from Faron Pharmaceuticals; payment for a lecture from Janssen; and payment for participation in advisory board of Astra Zeneca. Marianne Gunell: payments for lectures from Biocodex and Labquality, and a travel grant for ECCMID2019 from Turun Mikrobiologien Tiedesäätiö. Eveliina Munukka: payment for honoraria lectures ×3 regarding Microbiota and Health given to HCPs in Educational Evening events. Claes Ohlsson: grants to institution from the Swedish Research Council, the Swedish State under the agreement between the Swedish government and the county councils, the ALF-agreement, the Torsten Soderberg Foundation, the Novo Nordisk Foundation, and the Knut and Alice Wallenberg Foundation, and two patent applications in the fields of probiotics and bone health. Matti Poutanen: grants/contracts from Cancerfonden and Vetenskapsrådet in University of Gothenburg, Sweden. Pekka Taimen: research grant from Finnish Cancer Foundation and data management committee member in ProScreen Prostate Cancer Screening Trial.

  ***Funding/Support and role of the sponsor:*** This work was supported by grants from the Cancer Foundation Finland, TYKS-SAPA research fund of Turku University Hospital, and Finnish Cultural Foundation.

  ***Acknowledgments*:** The authors thank Heidi Isokääntä, Katri Kylä-Mattila, and Minna Lamppu for their excellent technical assistance in this study, and Peter B. Dean, MD D. Med. Sci. (University of Turku, Turku, Finland) for helping with the manuscript revision.

  ***Data sharing statement*:** All de-identified MRI data and the study protocol are available on the Multi-IMPROD site (http://petiv.utu.fi/multiimprod). All microbiological data will be available for bona fide researchers who request it from the authors.
