# Supplementary material for: Differences in Gut Microbiota Profiles and Microbiota Steroid Hormone Biosynthesis in Men with and Without Prostate Cancer
Source: Eur Urol Open Sci. 2024 Mar 13;62:140–50. doi: 10.1016/j.euros.2024.02.004 (PMC10946286; doi:10.1016/j.euros.2024.02.004)
Supplement: Supplementary data 1 [file mmc1.docx]

**Supplementary Appendix**

This appendix has been provided by the authors to give readers additional information about their work.

# Supplement to: (Kalinen S, Kallonen T, Gunell M, et al. Differences in gut microbiota profiles and microbiota steroid hormone biosynthesis in men with and without prostate cancer)

**Table of Contents**

List of investigators3

Supplementary results4

Supplementary figures5

Supplementary Figure 15

Supplementary Figure 27

Supplementary Figure 38

Supplementary Figure 49

Supplementary Figure 510

Supplementary Figure 6A11

Supplementary Figure 6B12

Supplementary Figure 6C13

Supplementary Figure 714

Supplementary Figure 8A–I15

Supplementary Figure 9A–I16

Supplementary Tables17

Supplementary Table 117

Supplementary Table 219

**LIST OF INVESTIGATORS**

Sofia Kalinen^1,2^,B.M., Teemu Kallonen^2,3^,Ph.D., Marianne Gunell^2,3^,Ph.D., Otto Ettala^4^, M.D., Ph.D., Ivan Jambor^5^, M.D., Ph.D., Juha Knaapila^4^, M.D., Ph.D., Kari T. Syvänen^4^, M.D., Ph.D., Pekka Taimen^6,7^, M.D., Ph.D., Matti Poutanen^6,8,9^, Ph.D., Claes Ohlsson^9^, M.D., Ph.D., Hannu J. Aronen^5^, M.D., Ph.D., Helena Ollila^10^, M.Sc, Sami Pietilä^11^,M. Sc., Laura L. Elo^6,11^, Ph.D., Tarja Lamminen^4^, Ph.D., Antti J. Hakanen^2,3^, M.D., Ph.D., Eveliina Munukka^3,12^, Ph.D., Peter J. Boström^4^, M.D., Ph.D., and the Multi-IMPROD Study group*

^1^ Research Center for Infections and Immunity, Institute of Biomedicine, University of Turku, Turku, Finland

^2^Department of Clinical Microbiology, Turku University Hospital, Turku, Finland

^3^Clinical Microbiome Bank, Microbe Center, Turku University Hospital and University of Turku, Turku, Finland
^4^Department of Urology, Turku University Hospital and University of Turku, Turku, Finland
^5^Department of Radiology, Turku University Hospital and University of Turku, Turku, Finland
^6^Institute of Biomedicine, University of Turku, Turku, Finland
^7^Department of Pathology, Turku University Hospital, Turku, Finland
^8^Centre for Integrative Physiology and Pharmacology, University of Turku, Turku, Finland
^9^Department of Internal Medicine and Clinical Nutrition, Institute of Medicine, Sahlgrenska Academy, University of Gothenburg, Gothenburg, Sweden
^10^Turku Clinical Research Centre, Turku University Hospital, Turku, Finland
^11^Turku Bioscience Centre, University of Turku and Åbo Akademi University, Turku, Finland
^12^Biocodex: Biocodex Nordics, Espoo, Finland

***Multi-IMPROD study group:**

From the Multi-IMPROD study group, Included in author list: Ivan Jambor, Otto Ettala, Juha Knaapila, Pekka Taimen, Kari T. Syvänen, Tarja Lamminen, Hannu Aronen, Peter J. Boström

The study group also includes following investigators:

Janne Verho, Department of Radiology, Turku University Hospital and University of Turku, Turku, Finland

Aida Steiner, Department of Radiology, Turku University Hospital and University of Turku, Turku, Finland

Esa Kähkönen, Department of Urology, Turku University Hospital and University of Turku, Turku, Finland

Ileana Montoya Perez, Department of Computing, University of Turku, Turku, Finland

Marjo Seppänen, Department of Surgery, Satakunta Central Hospital, Pori, Finland

Antti Rannikko, Department of Urology, Helsinki University, and Helsinki University Hospital, Helsinki Turku, Finland

Outi Oksanen, Department of Radiology, Helsinki University Hospital, Helsinki, Finland

Jarno Riikonen, Department of Urology, Tampere University Hospital, Tampere, Finland

Sanna-Mari Vimpeli, Department of Radiology, Tampere University Hospital, Tampere, Finland

Harri Merisaari, Department of Radiology, University of Turku

Markku Kallajoki, Department of Pathology, Turku University Hospital

Tuomas Mirtti, Department of Pathology, University of Helsinki, Helsinki, Finland

Jani Saunavaara, Department of Radiology, University of Turku, Turku Finland

**SUPPLEMENTARY RESULTS**

**Sample quality and sequencing batch effect**

β-diversity measures and principal coordinate plots can be used to assess quality. In this study all used metrics showed eight diverging samples in principal coordinate plot (**Supplementary** **Fig. 1A–C**). Those eight samples had been extracted in the same batch.

Sequencing batch effect is seen on the PCo1 (18%) (**Supplementary** **Fig. 1C**). The batch effect was not seen on Weighted Uni Frac nor Bray-Curtis (**Supplementary** **Fig. 1A and 1B**). Unweighted Uni Frac describes the absence/presence of microbes in the sample whereas weighted Uni Frac and Bray Curtis dissimilarity also take the abundance into account. According to these results the use of Weighted Uni-Frac and Bray-Curtis is acceptable.

**Rarefaction curves**

Number of reads and the Chao1 and Shannon entropies of the samples. The rarefaction level 10527 was set for the analyses as all the samples could meet that level (**Supplementary** **Fig. 2**).

**α-Diversity**

Shannon index and Chao1 metric did not show significant differences between the groups (**Supplementary** **Fig. 3 and 4**).

**β-Diversity**

Bray-Curtis dissimilarity between ISUP Grade Groups in principal coordinate plot. Statistical significance was tested with PERMANOVA with 99999 permutations. There were no significant differences between ISUP grade groups (**Supplementary** **Fig. 5).**

**Differential abundance analysis**

Table of significantly differentially abundant genera between cancer and benign cases with taxonomy information is presented in **Supplementary Table 1.** This was not a follow up study. However, differential abundance analysis was performed with ISUP grade groups to find out the microbiota associated with the prostate cancer severity. Several bacterial genera trend in the similar manner across the grade groups. The bacteria genera could be divided to initial, lower abundance in cancer and higher abundance in cancer according to log_2_ fold changes compared to benign (**Supplementary** **Fig. 6A–C**). Paired Log_2_ fold changes between the cancer grade versus benign were calculated for the significant (FDR-corrected) genera in ANOVA-like test.

**Plasma steroid hormones assay**

To validate the hormonal assay method, the 5-α-reductase inhibitor (5-ARI) users (n=17) were compared to non-users (n=148). DHT was significantly lower (P<0.001) in 5-ARI users compared to non-users, as expected (**Supplementary** **Fig. 7**). Testosterone was statistically significantly lower with higher predicted microbial steroid hormone biosynthesis (P=0.03) There were no other significant differences in plasma levels according to predicted microbial steroid hormone biosynthesis (P>0.05) (**Supplementary** **Fig. 8A–H**). There were no significant differences in plasma steroid hormone levels according to cancer status (P>0.05) (**Supplementary** **Fig. 9A–H**).

**PICRUSt**

PICRUSt KEGG Pathways within P<0.10 are shown in the **Supplementary Table 2**.

**SUPPLEMENTARY FIGURES**

**Supplementary Figure 1**


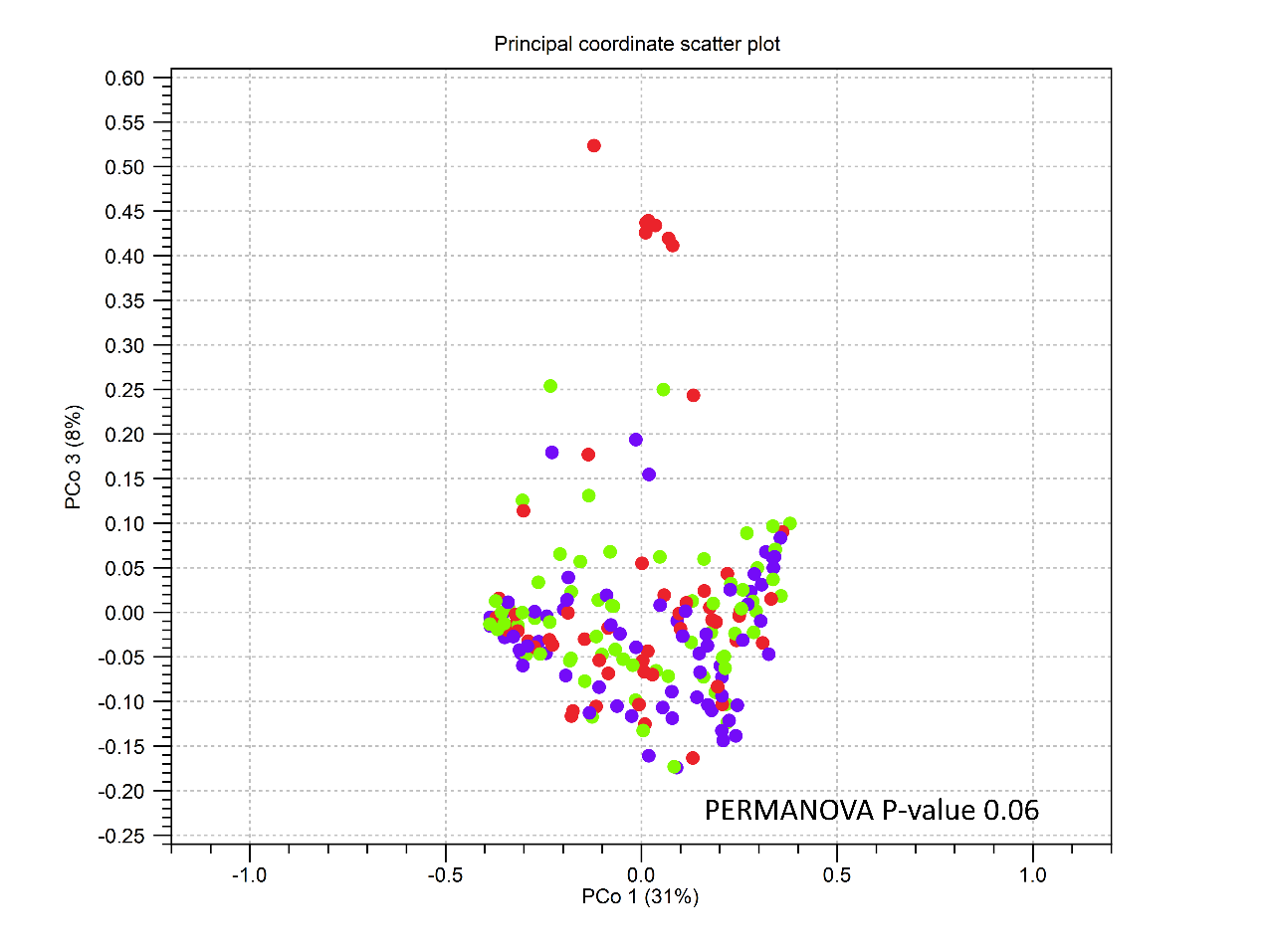


**A**


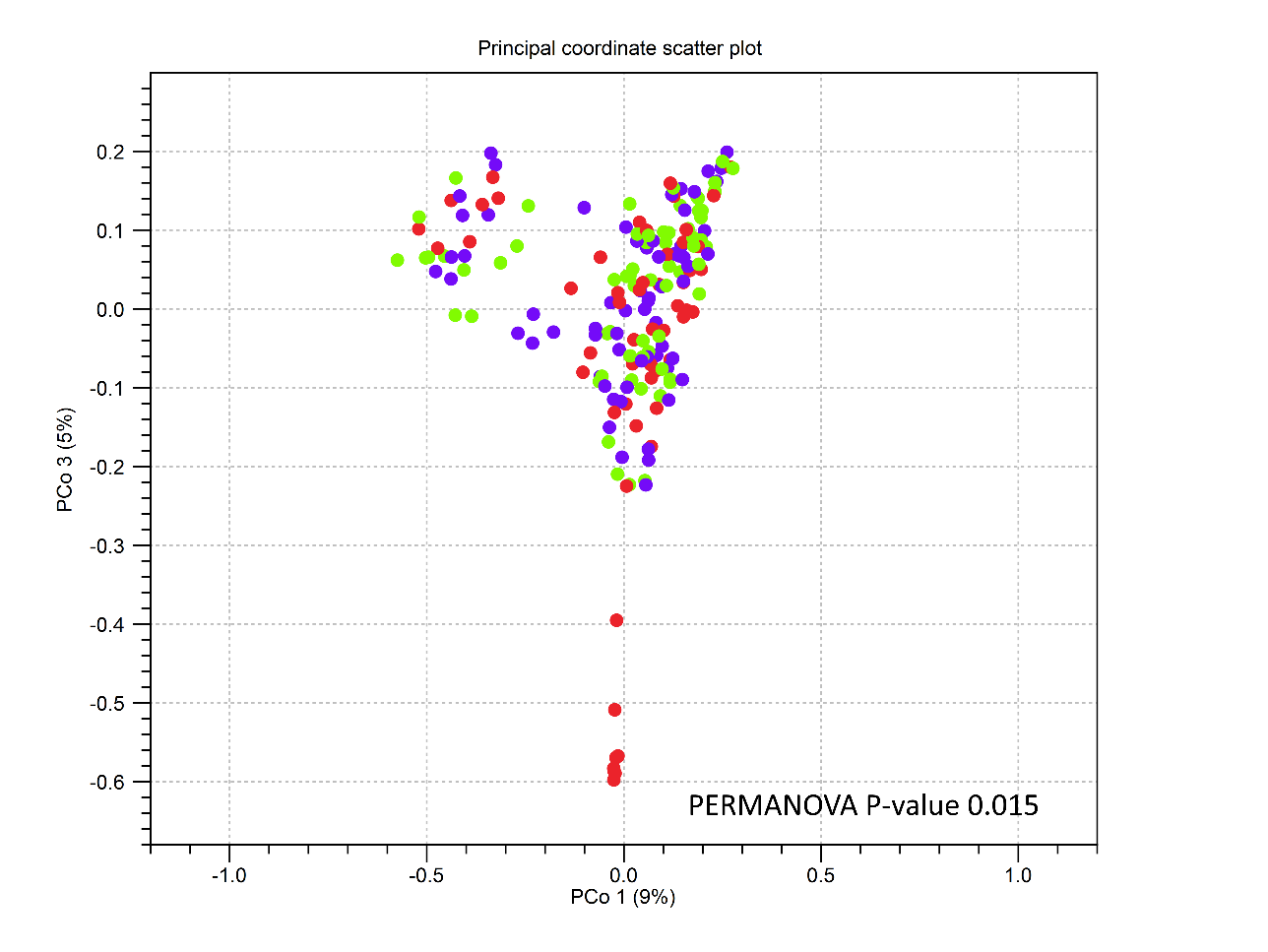


**B**


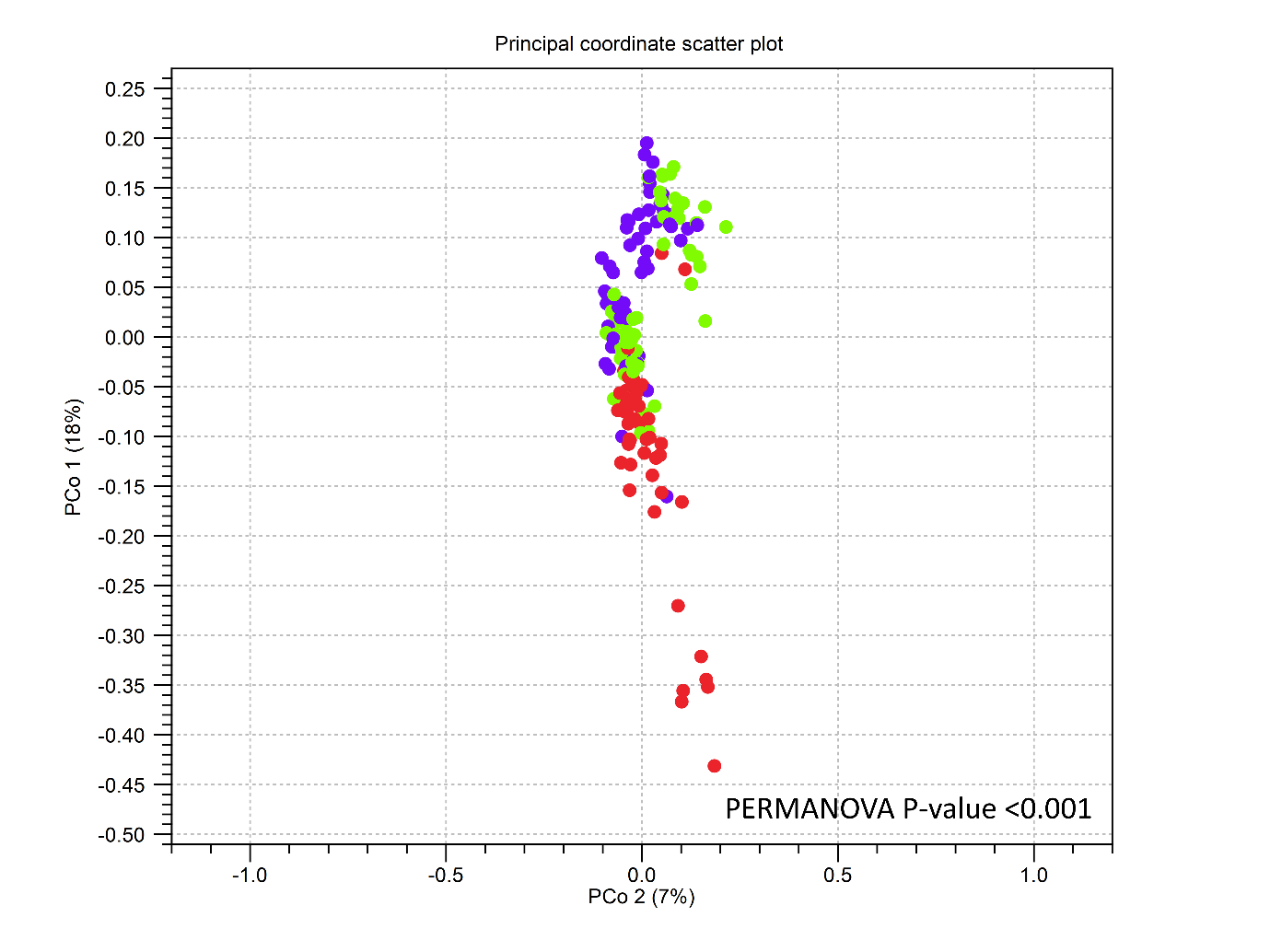


**C**

**Supplementary** **Figure 1 Sample quality and sequencing batch in principal coordinate plots. A) Weighted Uni Frac B) Bray-Curtis. C) Unweighted Uni Frac. Samples were sequenced in three batches (red, green, purple). 8 samples (red) did not meet the quality standards. These samples were in the same DNA extraction batch and were removed from the further analyses.**

**Supplementary** **Figure 2**


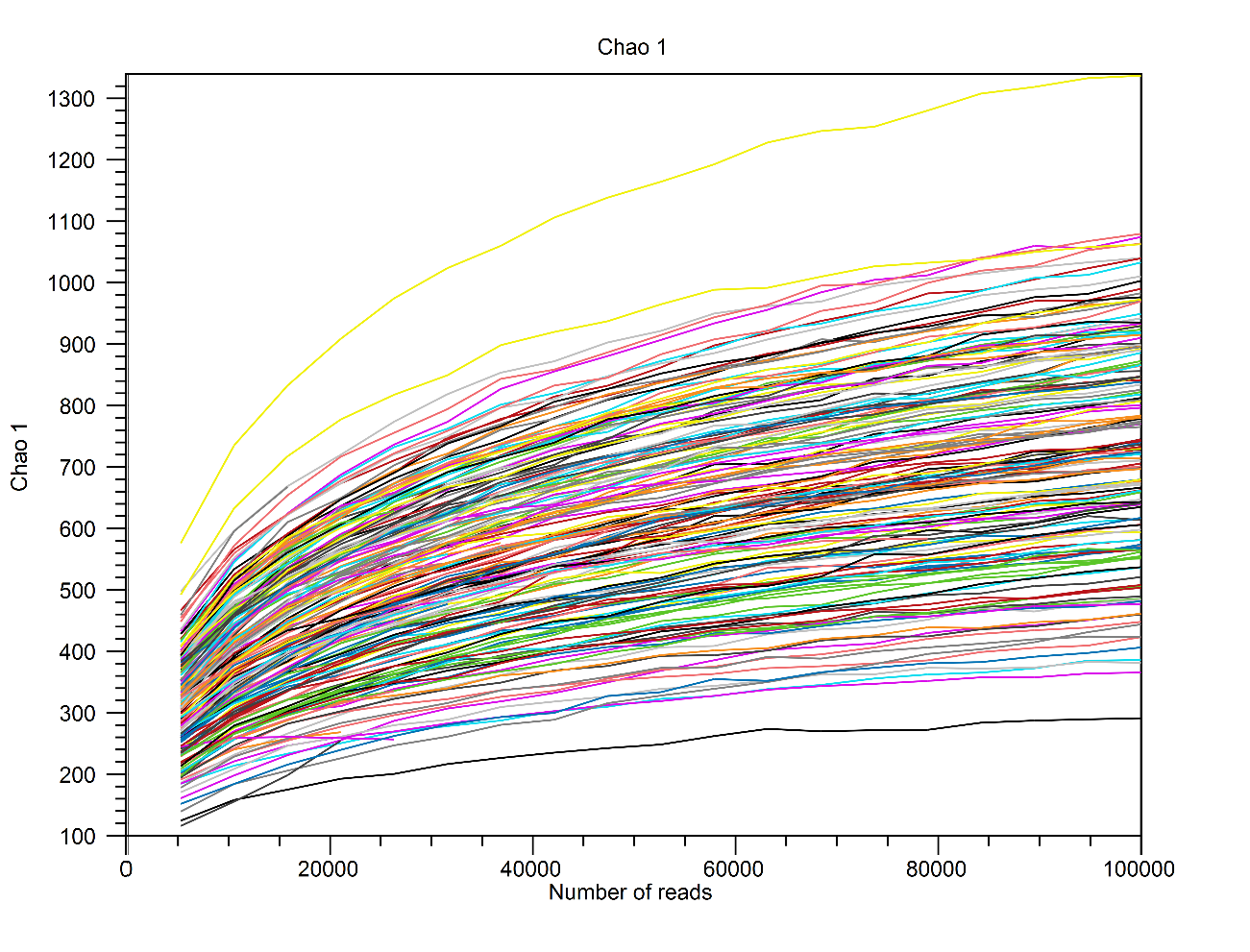

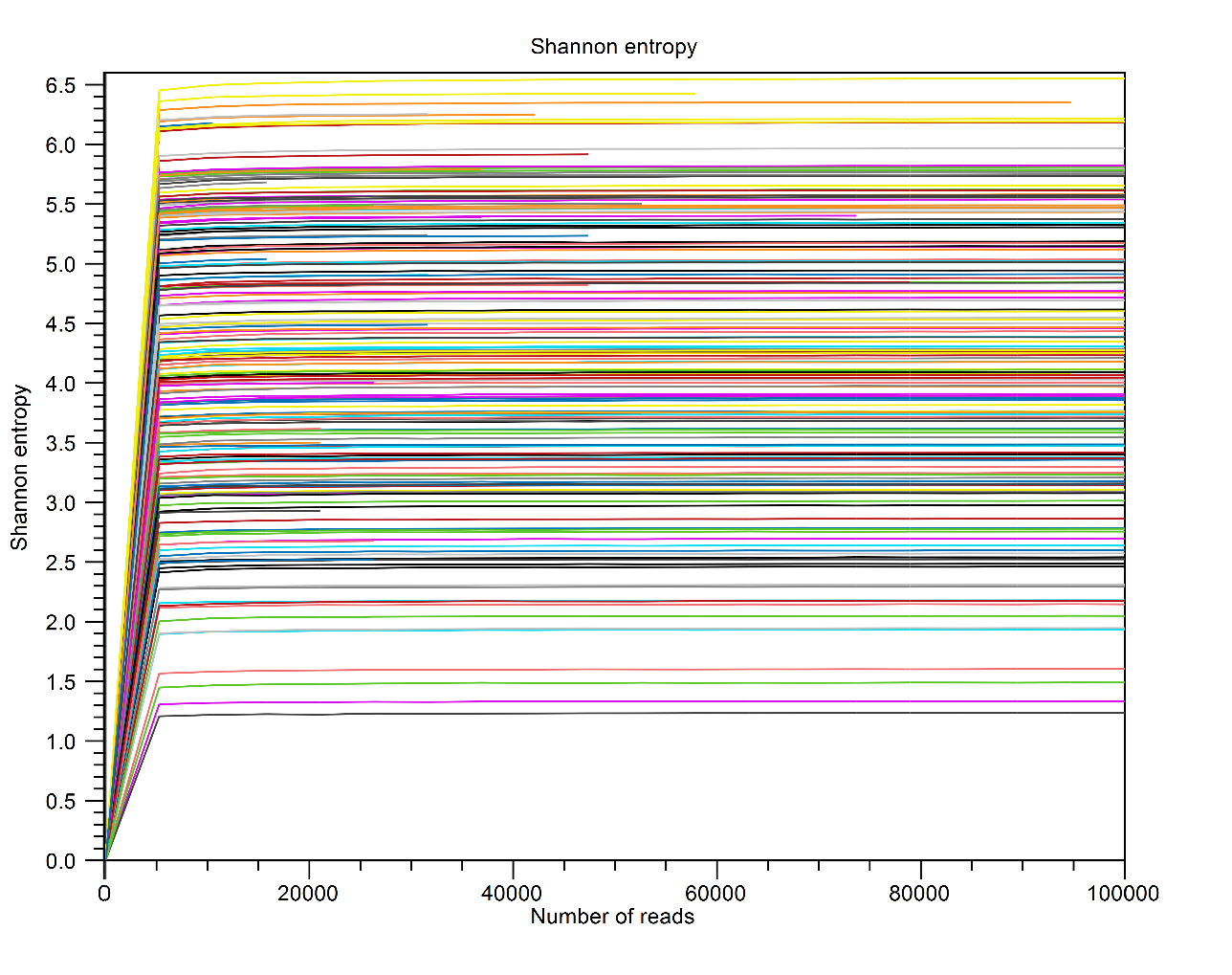


**B**

**A**

**Supplementary** **Figure 2 Number of reads and α-diversity indices A) Chao1 B) Shannon entropy.**

**Supplementary** **Figure 3**


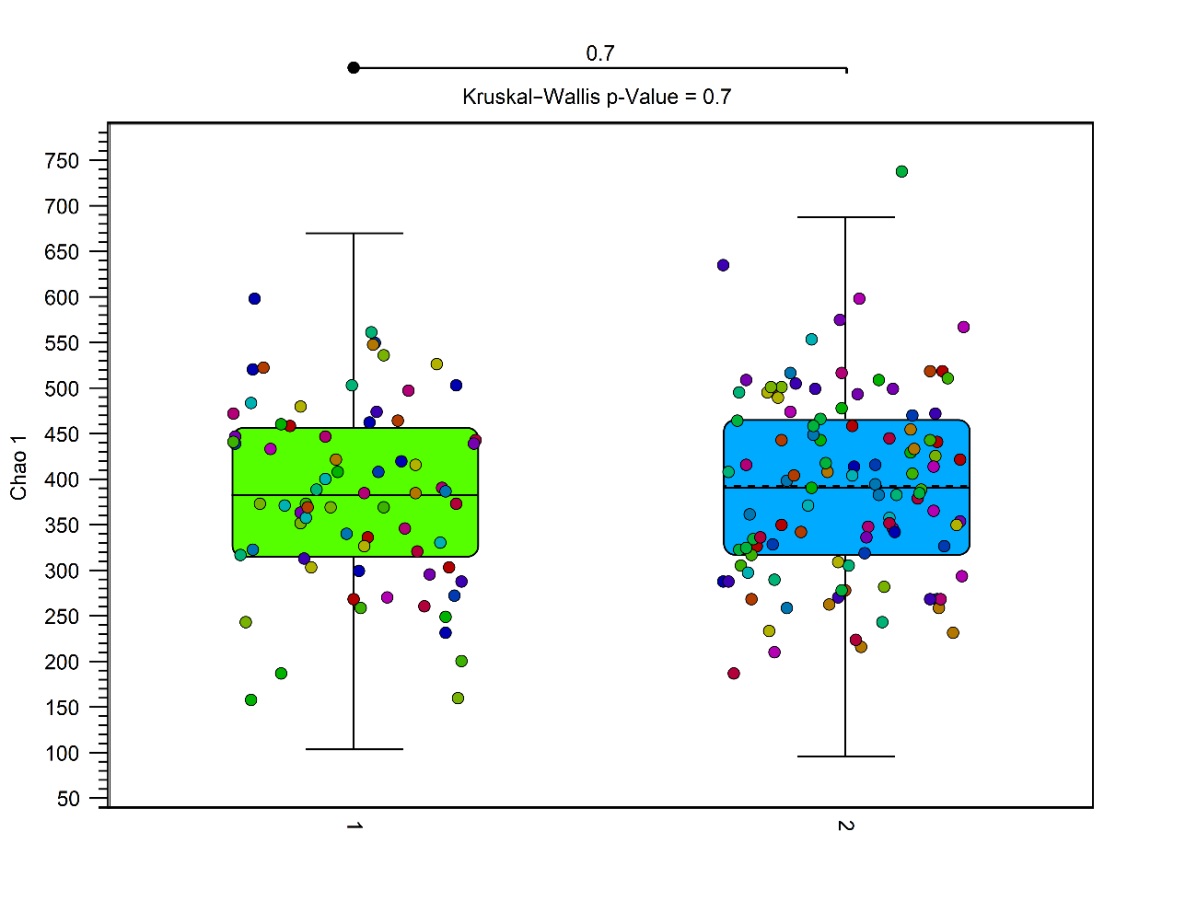

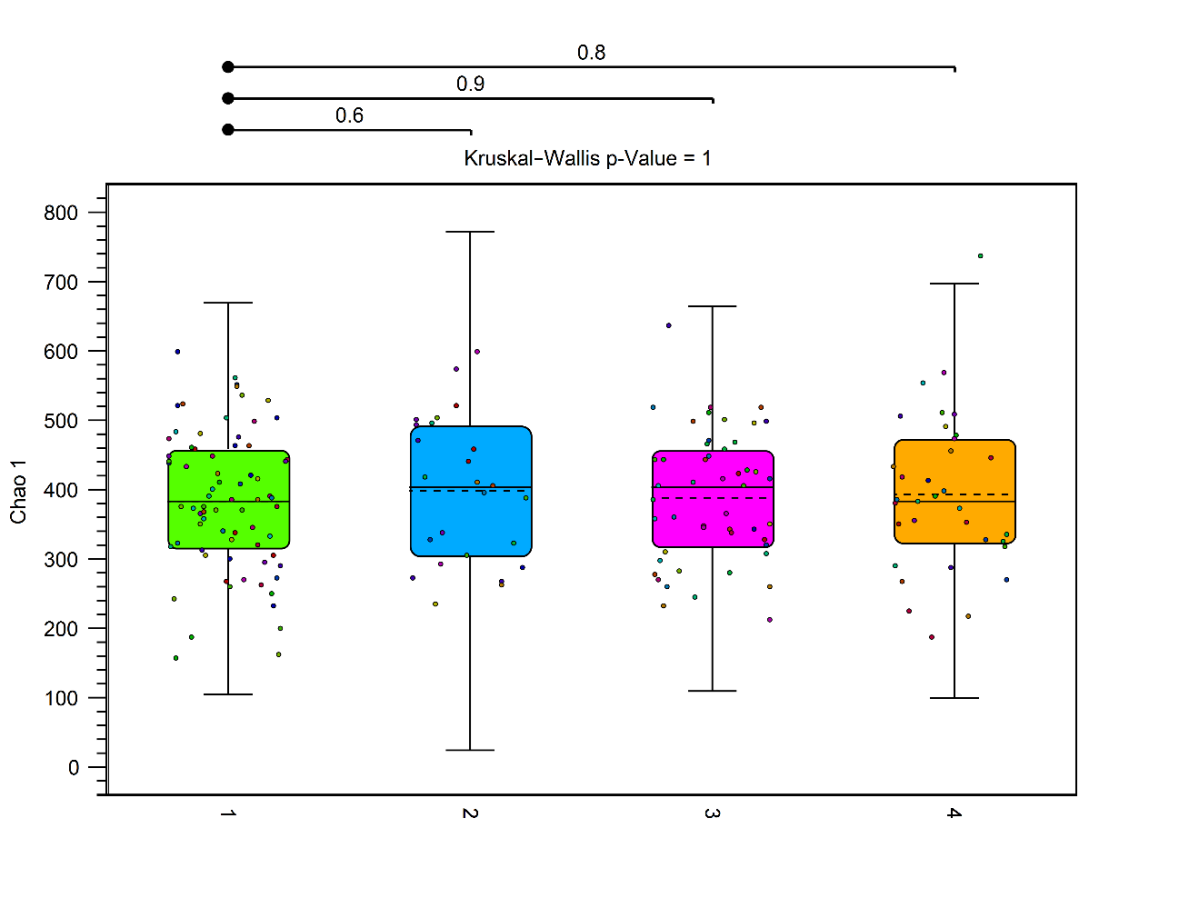


Benign

ISUP Grade 4-5

ISUP Grade 2-3

ISUP Grade 1

Prostate cancer

Benign

**B**

**A**

**Supplementary** **Figure 3 α-diversity of study groups. A) Chao1 compared between benign and prostate cancer. B) Chao1 compared between benign and ISUP grade groups. There were no significant differences between benign and prostate cancer nor across the cancer severity (Wilcoxon rank sum test, Kruskal-Wallis test).**

**Supplementary** **Figure 4**


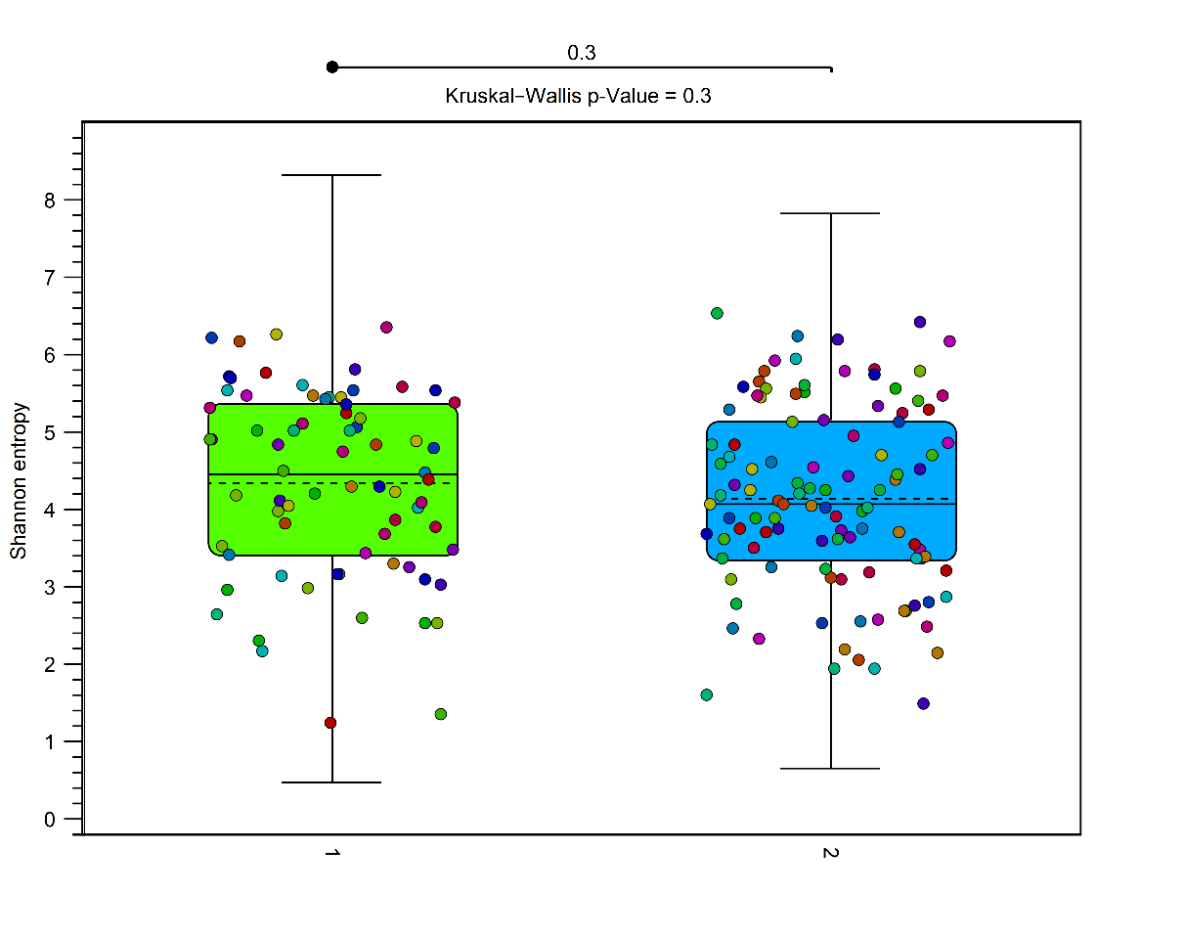

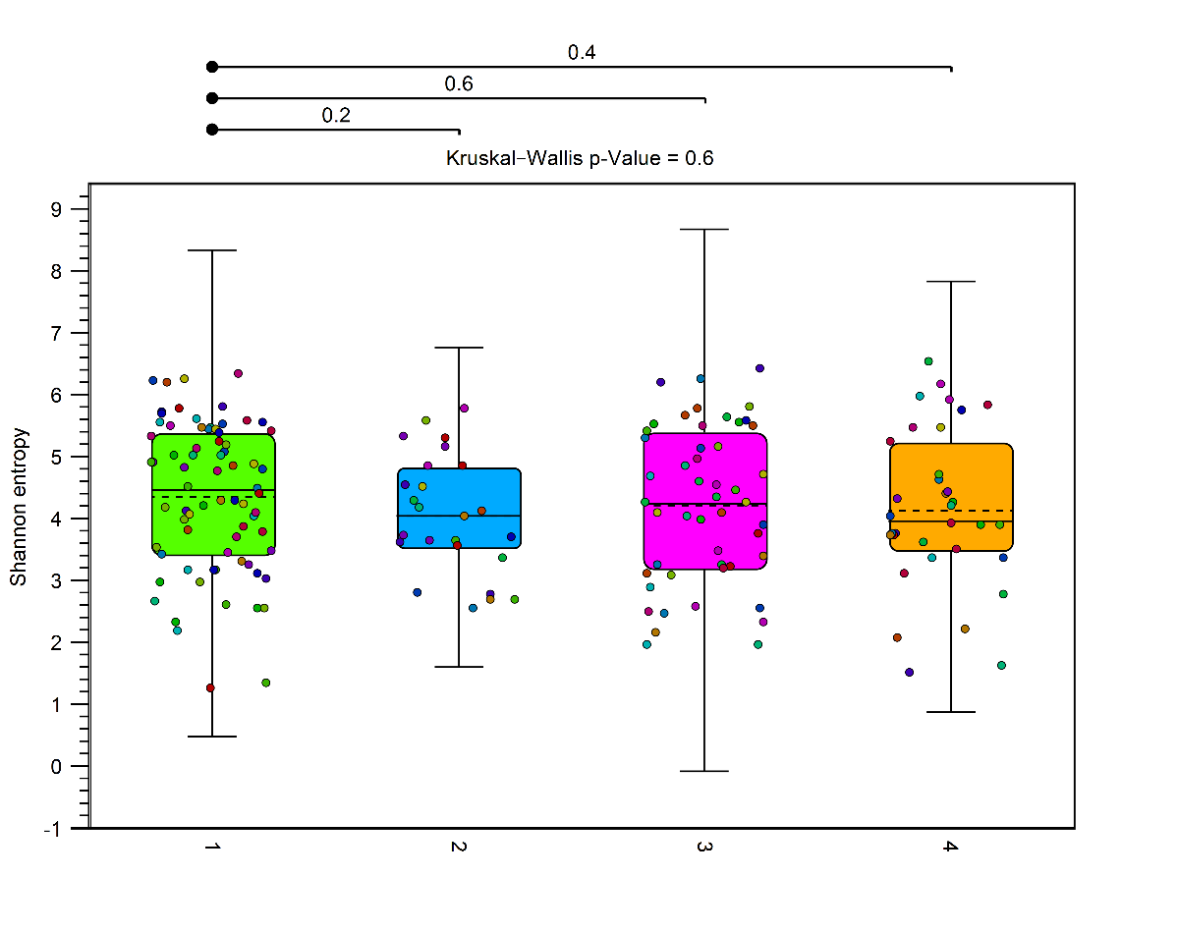


Prostate cancer

Benign

ISUP Grade 4-5

ISUP Grade 2-3

ISUP Grade 1

Benign

**B**

**A**

**Supplementary** **Figure 4 α-diversity of study groups. A) Shannon entropy compared between benign and prostate cancer. B) Shannon entropy compared between benign and ISUP grade groups. There were no significant differences between benign and prostate cancer nor across the cancer severity (Wilcoxon rank sum test, Kruskal-Wallis test).**

**Supplementary** **Figure 5**


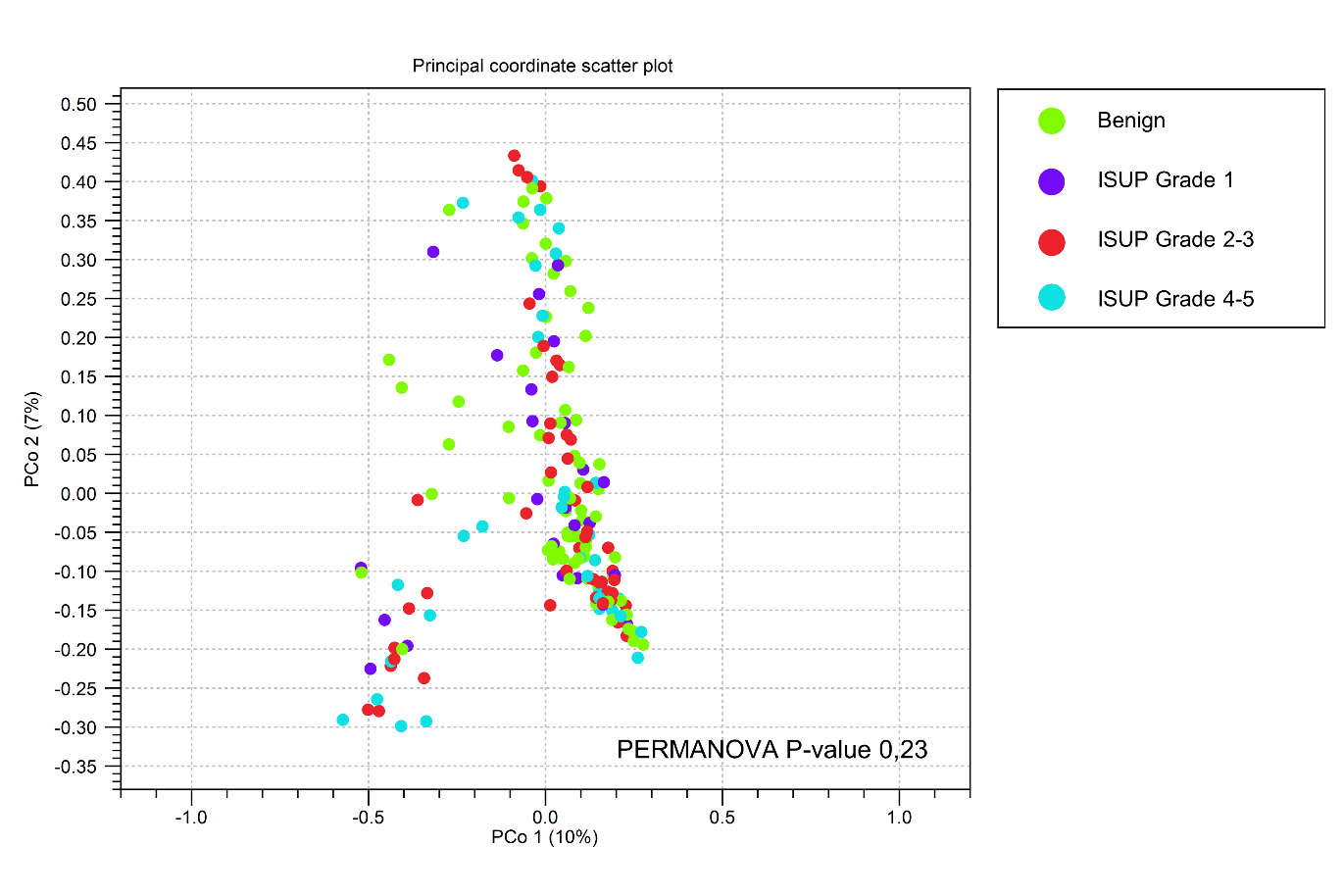


**Supplementary** **Figure 5 β-Diversity** **of ISUP Grade Groups in principal coordinate plot. Prostate cancer grade is shown in different colors: green for benign, purple for ISUP Grade 1, red for ISUP Grade 2-3 and blue for ISUP grade 4-5 (the most severe prostate cancer). There were no significant differences in the diversity between the ISUP grade groups.**

**Supplementary** **Figure 6**


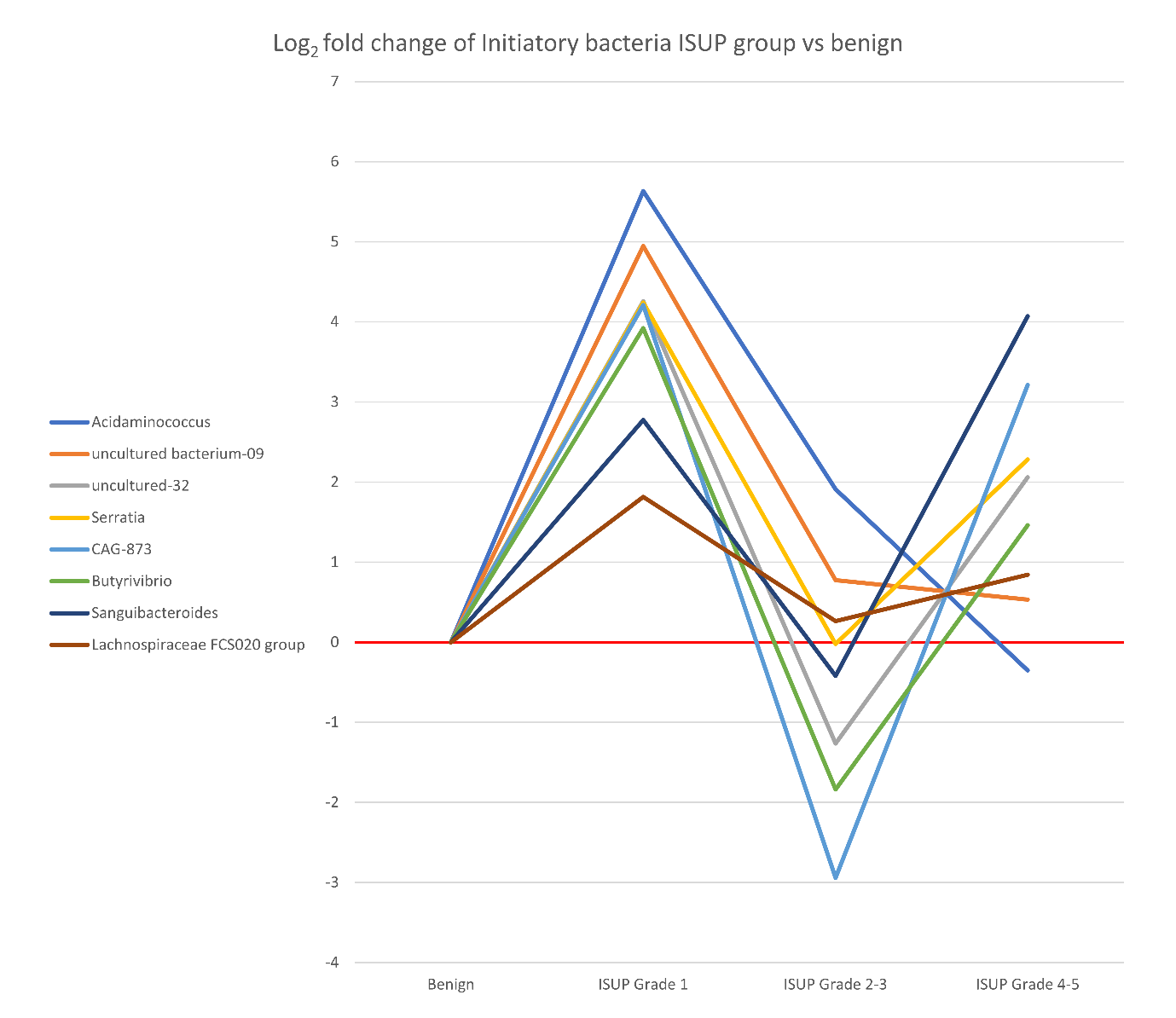


**A**

**Supplementary** **Figure 6A Log_2_ fold changes of potentially cancer initiating bacteria (most changes in the ISUP Grade 1 group) according to ISUP grade groups. Every color represents different bacteria genus across the cancer severity according to ISUP grade groups.**


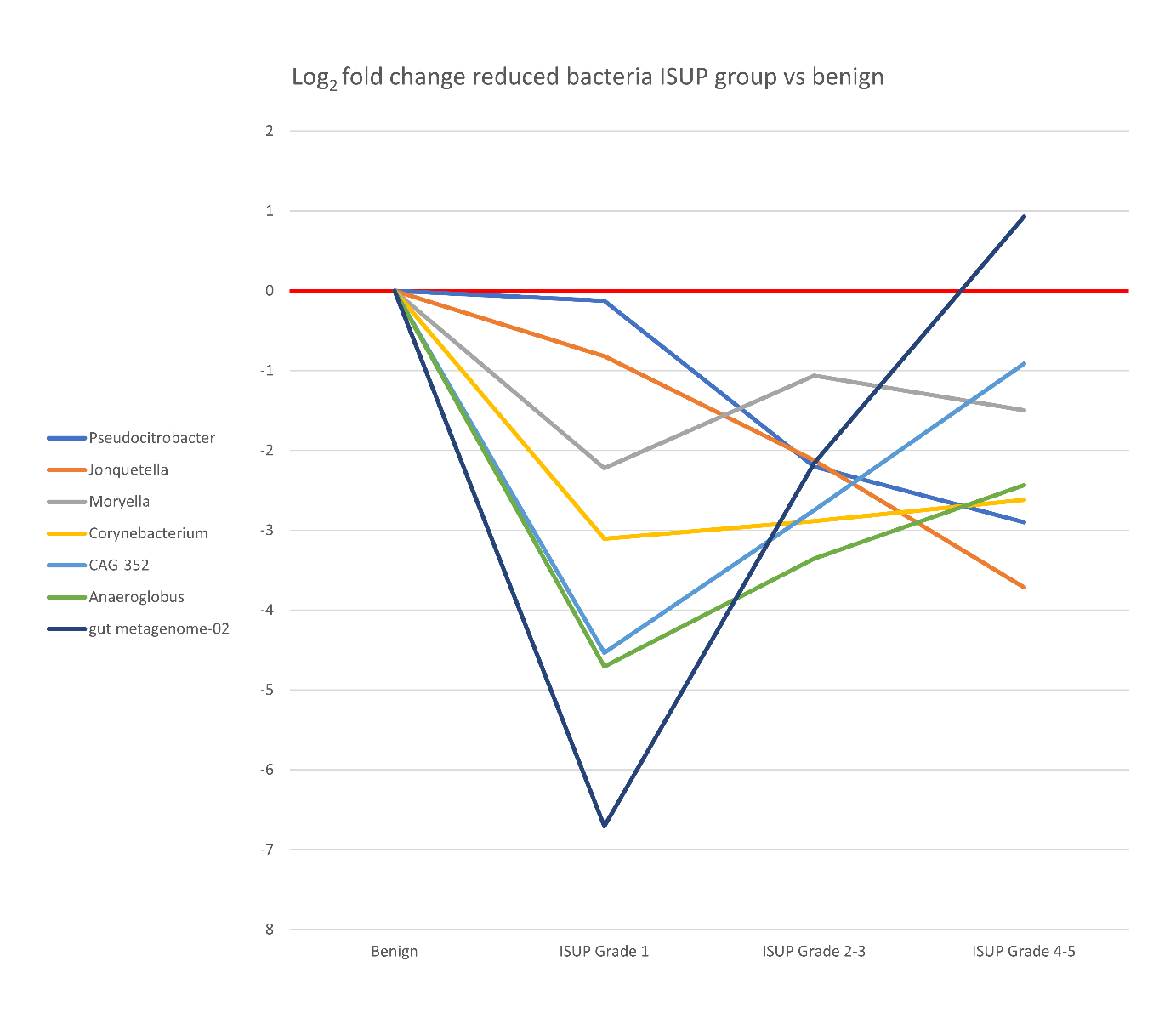
 **Supplementary Figure 6B Log_2_ fold changes of lower abundance bacteria in prostate cancer according to ISUP grade groups. Every color represents different bacteria genus across the cancer severity according to ISUP grade groups.**
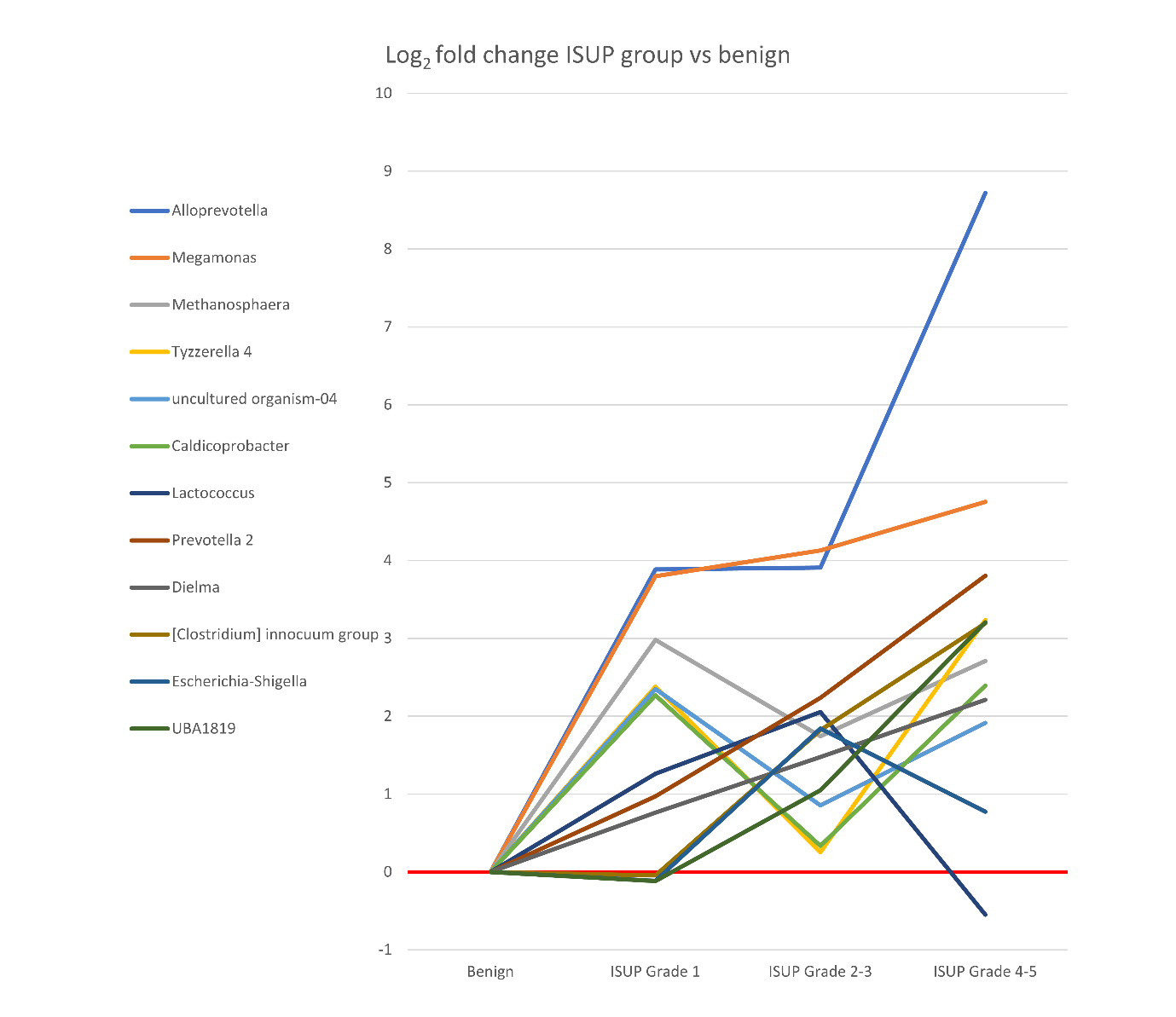
**Supplementary Figure 6C Log_2_ fold changes of higher abundance bacteria in prostate cancer according to ISUP grade groups. Every color represents different bacteria genus across the cancer severity according to ISUP grade groups.**

**B**

**B**

**C**

**Supplementary** **Figure 7**


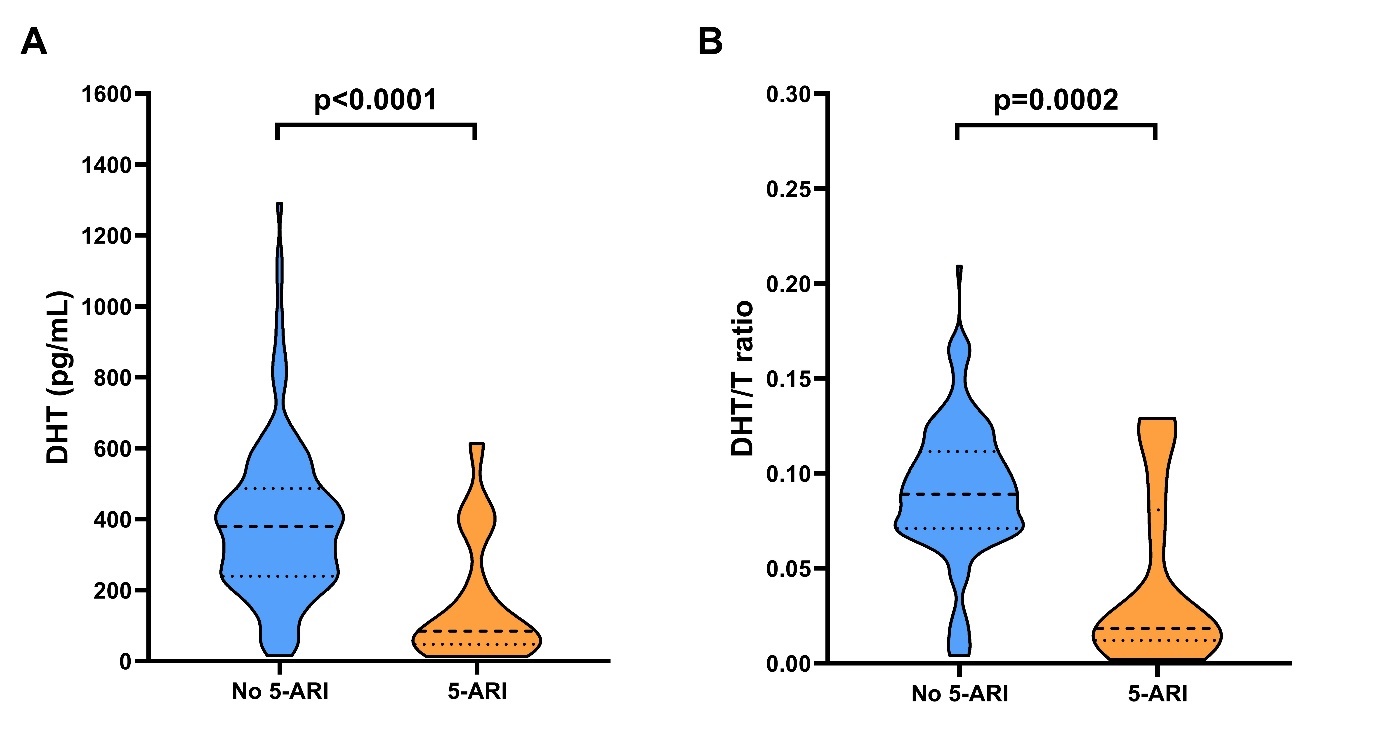


**Supplementary Figure 7. A. DHT concentration (pg/mL) in plasma by 5-α-reductase inhibitor (5-ARI) medication status. Among 5-ARI users (n=17), DHT levels are significantly lower in plasma compared to patients without 5-ARI medication (n=148, Wilcoxon Rank Sum test). B. DHT/T ratio in plasma by 5-ARI medication status. Among 5-ARI users (n=17), DHT/T ratio is significantly lower in plasma compared to patients without 5-ARI medication (n=148, Wilcoxon Rank Sum Test).**

**Supplementary**
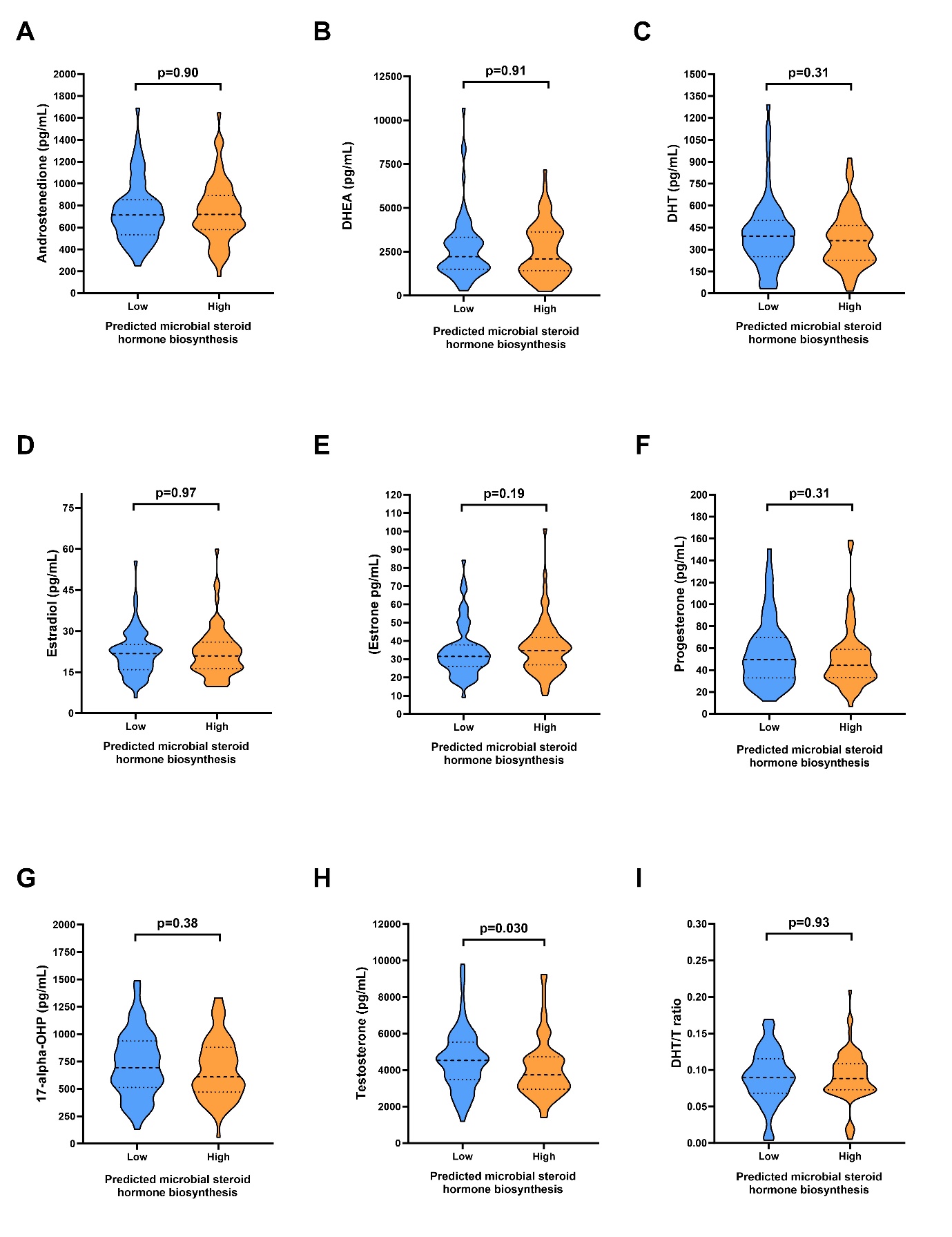
**Figure 8**

**Supplementary Figure 8A–H Plasma steroid hormones according to predicted microbial steroid hormone biosynthesis.** **A) Androstenedione, B) DHT, C) DHEA, D) Estradiol, E) Estrone, F) Progesterone, G) 17-α-hydroxyprogesterone (17-alpha-OHP), H) Testosterone (pg/mL), and I) DHT/T ratio according to predicted microbial steroid hormone biosynthesis. Testosterone was statistically significantly lower with higher predicted microbial steroid hormone biosynthesis (P=0.030) There were no other significant differences in plasma levels according to predicted microbial steroid hormone biosynthesis (P>0.05).**

**Supplementary**
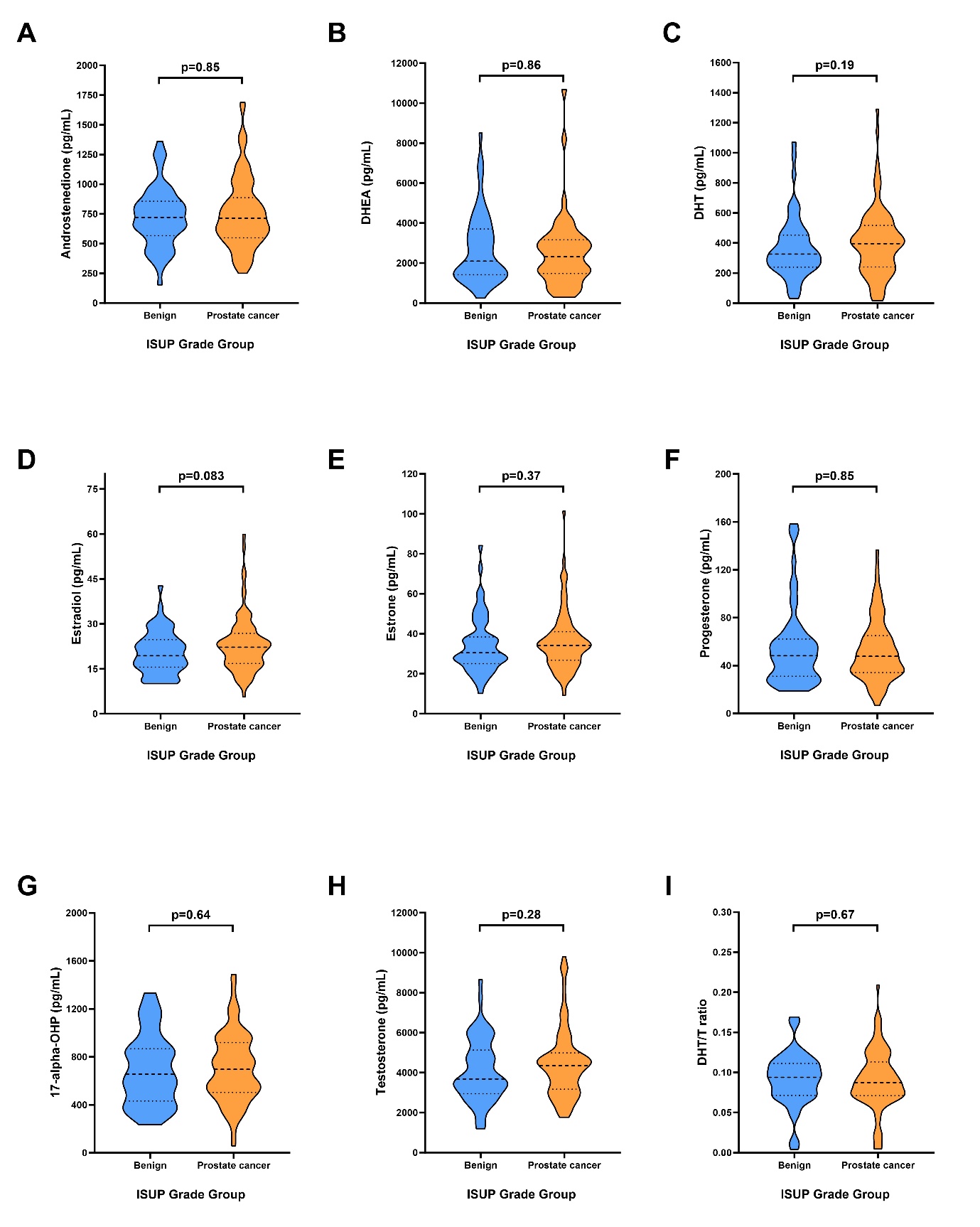
**Figure 9**

**Supplementary** **Figure 9A–H Plasma steroid hormones according to prostate cancer status. A) Androstenedione, B) DHT, C) DHEA, D) Estradiol, E) Estrone, F) Progesterone, G) 17-α-hydroxyprogesterone (17-alpha-OHP), H) Testosterone (pg/mL), and I) DHT/T ratio according to prostate cancer status (ISUP Grade Group). There were no significant differences in plasma levels according to predicted microbial steroid hormone biosynthesis (P>0.05).**

**SUPPLEMENTARY TABLES**

**Supplementary Table 1 Differential abundance analysis significant results, combined abundance >100, prevalence >10%, FDR-corrected with taxonomy**

| **Taxonomy** | **Combined Abundance** | **Prevalence in samples (%)** | **Median of present samples** | **Prostate cancer vs Benign** | | | | |
| --- | --- | --- | --- | --- | --- | --- | --- | --- |
| **D_0 (Kingdom) D_1 (Phylum) D_2 (Class) D_3 (Order) D_4 (Family) D_5 (Genus)** |  |  |  | **Max group mean** | **Log₂ fold change** | **Fold change** | **P-value** | **FDR p-value** |
| D_0__Archaea, D_1__Euryarchaeota, D_2__Methanobacteria, D_3__Methanobacteriales, D_4__Methanobacteriaceae, D_5__Methanosphaera | 924 | 13 | 6 | 8 | 2.5 | 5.5 | <0.001 | 0.001 |
| D_0__Bacteria, D_1__Actinobacteria, D_2__Actinobacteria, D_3__Corynebacteriales, D_4__Corynebacteriaceae, D_5__Corynebacterium | 1359 | 35 | 4 | 13 | -2.9 | -7.5 | <0.001 | <0.001 |
| D_0__Bacteria, D_1__Bacteroidetes, D_2__Bacteroidia, D_3__Bacteroidales, D_4__Barnesiellaceae, D_5__uncultured-08 | 2665 | 17 | 39 | 20 | 2.1 | 4.2 | 0.001 | 0.009 |
| D_0__Bacteria, D_1__Bacteroidetes, D_2__Bacteroidia, D_3__Bacteroidales, D_4__Marinifilaceae, D_5__Sanguibacteroides | 1124 | 12 | 25 | 9 | 2.1 | 4.2 | 0.001 | 0.009 |
| D_0__Bacteria, D_1__Bacteroidetes, D_2__Bacteroidia, D_3__Bacteroidales, D_4__Muribaculaceae, D_5__CAG-873 | 92781 | 24 | 4 | 678 | 2.3 | 4.8 | 0.001 | 0.01 |
| D_0__Bacteria, D_1__Bacteroidetes, D_2__Bacteroidia, D_3__Bacteroidales, D_4__Muribaculaceae, D_5__uncultured organism-02 | 976 | 13 | 1 | 6 | 2.9 | 7.4 | <0.001 | 0.002 |
| D_0__Bacteria, D_1__Bacteroidetes, D_2__Bacteroidia, D_3__Bacteroidales, D_4__Prevotellaceae, D_5__Alloprevotella | 573293 | 50 | 8 | 4625 | 5.5 | 45.6 | <0.001 | <0.001 |
| D_0__Bacteria, D_1__Bacteroidetes, D_2__Bacteroidia, D_3__Bacteroidales, D_4__Prevotellaceae, D_5__Prevotella 2 | 15028 | 13 | 3 | 136 | 2.2 | 4.4 | 0.001 | 0.02 |
| D_0__Bacteria, D_1__Bacteroidetes, D_2__Bacteroidia, D_3__Bacteroidales, D_4__Prevotellaceae, D_5__Prevotella 9 | 4413629 | 100 | 244 | 30682 | 1.5 | 2.8 | 0.003 | 0.03 |
| D_0__Bacteria, D_1__Bacteroidetes, D_2__Bacteroidia, D_3__Sphingobacteriales, D_4__Lentimicrobiaceae, D_5__uncultured bacterium-09 | 11402 | 24 | 63 | 88 | 2.2 | 4.6 | 0.001 | 0.010 |
| D_0__Bacteria, D_1__Cyanobacteria, D_2__Melainabacteria, D_3__Gastranaerophilales, Ambiguous_taxa, Ambiguous_taxa-07 | 4671 | 16 | 12 | 34 | 2.5 | 5.8 | <0.001 | 0.002 |
| D_0__Bacteria, D_1__Cyanobacteria, D_2__Melainabacteria, D_3__Gastranaerophilales, D_4__gut metagenome, D_5__gut metagenome-02 | 9133 | 11 | 20 | 71 | -2.4 | -5.3 | <0.001 | 0.008 |
| D_0__Bacteria, D_1__Firmicutes, D_2__Bacilli, D_3__Lactobacillales, D_4__Streptococcaceae, D_5__Lactococcus | 2422 | 60 | 5 | 17 | 1.3 | 2.5 | 0.001 | 0.02 |
| D_0__Bacteria, D_1__Firmicutes, D_2__Clostridia, D_3__Clostridiales, D_4__Caldicoprobacteraceae, D_5__Caldicoprobacter | 406 | 23 | 1 | 3 | 1.7 | 3.2 | 0.001 | 0.01 |
| D_0__Bacteria, D_1__Firmicutes, D_2__Clostridia, D_3__Clostridiales, D_4__Christensenellaceae, D_5__Christensenellaceae R-7 group | 171030 | 98 | 175 | 1274 | 1.0 | 2.1 | 0.002 | 0.02 |
| D_0__Bacteria, D_1__Firmicutes, D_2__Clostridia, D_3__Clostridiales, D_4__Clostridiales vadinBB60 group, Ambiguous_taxa-13 | 33714 | 80 | 20 | 295 | 1.6 | 3.1 | <0.001 | 0.002 |
| D_0__Bacteria, D_1__Firmicutes, D_2__Clostridia, D_3__Clostridiales, D_4__Clostridiales vadinBB60 group, D_5__uncultured organism-04 | 518 | 27 | 7 | 4 | 1.6 | 3.0 | 0.001 | 0.01 |
| D_0__Bacteria, D_1__Firmicutes, D_2__Clostridia, D_3__Clostridiales, D_4__Clostridiales vadinBB60 group, D_5__uncultured Thermoanaerobacterales bacterium | 2338 | 43 | 10 | 18 | 1.7 | 3.2 | 0.001 | 0.01 |
| D_0__Bacteria, D_1__Firmicutes, D_2__Clostridia, D_3__Clostridiales, D_4__Lachnospiraceae, D_5__Butyrivibrio | 204311 | 50 | 4 | 1584 | 2.1 | 4.3 | 0.001 | 0.01 |
| D_0__Bacteria, D_1__Firmicutes, D_2__Clostridia, D_3__Clostridiales, D_4__Lachnospiraceae, D_5__Lachnospiraceae FCS020 group | 7920 | 84 | 17 | 58 | 1.0 | 2.0 | 0.003 | 0.03 |
| D_0__Bacteria, D_1__Firmicutes, D_2__Clostridia, D_3__Clostridiales, D_4__Lachnospiraceae, D_5__Lachnospiraceae UCG-003 | 16360 | 31 | 21 | 106 | 2.4 | 5.4 | <0.001 | 0.003 |
| D_0__Bacteria, D_1__Firmicutes, D_2__Clostridia, D_3__Clostridiales, D_4__Lachnospiraceae, D_5__Moryella | 6991 | 85 | 13 | 61 | -1.5 | -2.7 | <0.001 | 0.001 |
| D_0__Bacteria, D_1__Firmicutes, D_2__Clostridia, D_3__Clostridiales, D_4__Lachnospiraceae, D_5__Tyzzerella 4 | 27590 | 28 | 8 | 237 | 1.8 | 3.5 | 0.004 | 0.03 |
| D_0__Bacteria, D_1__Firmicutes, D_2__Clostridia, D_3__Clostridiales, D_4__Ruminococcaceae, D_5__CAG-352 | 14571 | 19 | 7 | 125 | -3.3 | -9.8 | <0.001 | <0.001 |
| D_0__Bacteria, D_1__Firmicutes, D_2__Clostridia, D_3__Clostridiales, D_4__Ruminococcaceae, D_5__Hydrogenoanaerobacterium | 2547 | 43 | 7 | 22 | 2.0 | 4.0 | <0.001 | 0.001 |
| D_0__Bacteria, D_1__Firmicutes, D_2__Clostridia, D_3__Clostridiales, D_4__Ruminococcaceae, D_5__Oscillospira | 1155 | 22 | 4 | 7 | 1.5 | 2.8 | 0.006 | 0.05 |
| D_0__Bacteria, D_1__Firmicutes, D_2__Clostridia, D_3__Clostridiales, D_4__Ruminococcaceae, D_5__UBA1819 | 27061 | 92 | 18 | 193 | 1.1 | 2.2 | 0.005 | 0.04 |
| D_0__Bacteria, D_1__Firmicutes, D_2__Erysipelotrichia, D_3__Erysipelotrichales, D_4__Erysipelotrichaceae, D_5__[Clostridium] innocuum group | 466 | 23 | 3 | 4 | 2.1 | 4.2 | <0.001 | 0.002 |
| D_0__Bacteria, D_1__Firmicutes, D_2__Erysipelotrichia, D_3__Erysipelotrichales, D_4__Erysipelotrichaceae, D_5__Coprobacillus | 1284 | 17 | 6 | 10 | 4.4 | 20.7 | <0.001 | <0.001 |
| D_0__Bacteria, D_1__Firmicutes, D_2__Erysipelotrichia, D_3__Erysipelotrichales, D_4__Erysipelotrichaceae, D_5__Dielma | 167 | 21 | 3 | 1 | 1.5 | 2.9 | 0.001 | 0.01 |
| D_0__Bacteria, D_1__Firmicutes, D_2__Negativicutes, D_3__Selenomonadales, D_4__Acidaminococcaceae, D_5__Acidaminococcus | 112181 | 43 | 7 | 903 | 2.5 | 5.6 | <0.001 | 0.005 |
| D_0__Bacteria, D_1__Firmicutes, D_2__Negativicutes, D_3__Selenomonadales, D_4__Veillonellaceae, D_5__Anaeroglobus | 12425 | 44 | 7 | 141 | -3.0 | -7.8 | <0.001 | <0.001 |
| D_0__Bacteria, D_1__Lentisphaerae, D_2__Lentisphaeria, D_3__Victivallales, D_4__vadinBE97, D_5__uncultured rumen bacterium-04 | 528 | 22 | 8 | 4 | 1.5 | 2.8 | 0.004 | 0.04 |
| D_0__Bacteria, D_1__Proteobacteria, D_2__Gammaproteobacteria, D_3__Betaproteobacteriales, D_4__Burkholderiaceae, D_5__Alcaligenes | 700 | 54 | 3 | 4 | -1.2 | -2.2 | 0.003 | 0.03 |
| D_0__Bacteria, D_1__Proteobacteria, D_2__Gammaproteobacteria, D_3__Betaproteobacteriales, D_4__Burkholderiaceae, D_5__Parasutterella | 77911 | 90 | 44 | 495 | -1.3 | -2.5 | 0.002 | 0.02 |
| D_0__Bacteria, D_1__Proteobacteria, D_2__Gammaproteobacteria, D_3__Betaproteobacteriales, D_4__Neisseriaceae, D_5__uncultured-29 | 1206 | 12 | 17 | 10 | 2.5 | 5.5 | <0.001 | 0.002 |
| D_0__Bacteria, D_1__Proteobacteria, D_2__Gammaproteobacteria, D_3__Enterobacteriales, D_4__Enterobacteriaceae, Ambiguous_taxa-29 | 139 | 27 | 2 | 1 | 1.3 | 2.4 | 0.003 | 0.03 |
| D_0__Bacteria, D_1__Proteobacteria, D_2__Gammaproteobacteria, D_3__Enterobacteriales, D_4__Enterobacteriaceae, D_5__Citrobacter | 533 | 13 | 7 | 4 | 1.7 | 3.4 | 0.002 | 0.02 |
| D_0__Bacteria, D_1__Proteobacteria, D_2__Gammaproteobacteria, D_3__Enterobacteriales, D_4__Enterobacteriaceae, D_5__Escherichia-Shigella | 551481 | 98 | 229 | 3314 | 1.3 | 2.5 | 0.003 | 0.03 |
| D_0__Bacteria, D_1__Synergistetes, D_2__Synergistia, D_3__Synergistales, D_4__Synergistaceae, D_5__Jonquetella | 27709 | 40 | 14 | 293 | -2.4 | -5.2 | <0.001 | 0.001 |
| D_0__Bacteria, D_1__Verrucomicrobia, D_2__Verrucomicrobiae, D_3__Opitutales, D_4__Puniceicoccaceae, D_5__uncultured-32 | 4177 | 77 | 10 | 35 | 2.4 | 5.3 | <0.001 | 0.002 |

**Supplementary Table 2 PICRUSt results P<0.10**

| **KEGG Pathway** | **Benign** | | **Cancer** | | **p-value** |
| --- | --- | --- | --- | --- | --- |
|  | **Median** | **(IQR)** | **Median** | **(IQR)** |  |
| Mineral absorption | 2522 | (1197-8072) | 4044 | (1868-18695) | 0,008 |
| Steroid hormone biosynthesis | 8457 | (3373-16084) | 11717 | (4937-21186) | 0,022 |
| Retinol metabolism | 21605 | (8832-29630) | 23174 | (11812-35315) | 0,042 |
| Bladder cancer | 37 | (11-247) | 171 | (13-635) | 0,051 |
| Bacterial invasion of epithelial cells | 49 | (11-256) | 119 | (23-673) | 0,053 |
| African trypanosomiasis | 426 | (165-860) | 591 | (248-1252) | 0,057 |
| Fluorobenzoate degradation | 131 | (27-395) | 263 | (50-821) | 0,067 |
| Arachidonic acid metabolism | 37003 | (14831-69756) | 51649 | (19471-80097) | 0,073 |
| Carbohydrate digestion and absorption | 15218 | (5621-27855) | 21879 | (8317-33654) | 0,074 |
| Biosynthesis of siderophore group nonribosomal peptides | 30806 | (12789-35865) | 32048 | (24926-40301) | 0,083 |
| Glycan biosynthesis | 28853 | (15104-39625) | 33540 | (19369-41826) | 0,083 |
| Chagas disease (American trypanosomiasis) | 231 | (70-509) | 317 | (98-870) | 0,086 |
| Isoquinolone alcaloid biosynthesis | 59184 | (45862-71701) | 62282 | (45863-80307) | 0,096 |
